# The absence of thrombin-like activity in *Bothrops erythromelas* venom is due to the deletion of the snake venom thrombin-like enzyme gene

**DOI:** 10.1371/journal.pone.0248901

**Published:** 2021-04-27

**Authors:** Nicholas P. Lotto, Jeanne C. de Albuquerque Modesto, Sávio S. Sant’Anna, Kathleen F. Grego, Miriam C. Guarnieri, Rejane M. Lira-da-Silva, Marcelo L. Santoro, Nancy Oguiura

**Affiliations:** 1 Laboratory of Ecology and Evolution, Instituto Butantan, São Paulo, São Paulo, Brazil; 2 Vitória Academic Center, Federal University of Pernambuco, Vitória, Pernambuco, Brazil; 3 Laboratory of Herpetology, Instituto Butantan, São Paulo, São Paulo, Brazil; 4 Zoology Department, Federal University of Pernambuco, Recife, Pernambuco, Brazil; 5 Institute of Biology, Federal University of Bahia, Salvador, Bahia, Brazil; 6 Laboratory of Pathophysiology, Instituto Butantan, São Paulo, São Paulo, Brazil; Weizmann Institute of Science, ISRAEL

## Abstract

Snake venom thrombin-like enzymes (SVTLEs) are serine proteinases that clot fibrinogen. SVTLEs are distributed mainly in venoms from snakes of the Viperidae family, comprising venomous pit viper snakes. *Bothrops* snakes are distributed throughout Central and South American and are responsible for most venomous snakebites. Most *Bothrops* snakes display thrombin-like activity in their venoms, but it has been shown that some species do not present it. In this work, to understand SVTLE polymorphism in *Bothrop*s snake venoms, we studied individual samples from two species of medical importance in Brazil: *Bothrops jararaca*, distributed in Southeastern Brazil, which displays coagulant activity on plasma and fibrinogen, and *Bothrops erythromelas*, found in Northeastern Brazil, which lacks direct fibrinogen coagulant activity but shows plasma coagulant activity. We tested the coagulant activity of venoms and the presence of SVTLE genes by a PCR approach. The SVTLE gene structure in *B*. *jararaca* is similar to the *Bothrops atrox* snake, comprising five exons. We could not amplify SVTLE sequences from *B*. *erythromelas* DNA, except for a partial pseudogene. These genes underwent a positive selection in some sites, leading to an amino acid sequence diversification, mostly in exon 2. The phylogenetic tree constructed using SVTLE coding sequences confirms that they are related to the chymotrypsin/kallikrein family. Interestingly, we found a *B*. *jararaca* specimen whose venom lacked thrombin-like activity, and its gene sequence was a pseudogene with SVTLE structure, presenting nonsense and frameshift mutations. Our results indicate an association of the lack of thrombin-like activity in *B*. *jararaca* and *B*. *erythromelas* venoms with mutations and deletions of snake venom thrombin-like enzyme genes.

## Introduction

Snake venoms have many pharmacologically active toxins that disturb hemostasis, affecting blood platelet function, the coagulation cascade, and fibrinolysis. Snake venom toxins, which activate coagulation factors V, VIII, X, prothrombin, and fibrinogen, have been abundantly reported [1].

Fibrinogen is a 340-kDa glycoprotein present in plasma, which is essential for fibrin gel formation and platelet aggregation. Two symmetric units form it, consisting of three intertwined polypeptide chains (Aα, Bβ, and γ), dimerizing through a central globular region. Once fibrinopeptides A and B are cleaved from fibrinogen by thrombin, specific sites are exposed, and fibrin polymerization is initiated [2]. Among the various snake venom enzymes that act on fibrinogen, snake venom thrombin-like enzymes (SVTLEs) are serine proteinases akin to thrombin, as they may clot fibrinogen. Most SVTLEs cleave preferentially fibrinopeptides A or B, differently from thrombin that cleaves both peptides. SVTLE are present in snake venoms from the Viperidae family, comprising the genera *Agkistrodon*, *Bothrops*, *Protobothrops*, *Lachesis*, *Crotalus*, *Trimeresurus*, *Bitis*, and *Cerastes*, and they can be grouped in subgroups related to chymotrypsin, a pancreatic serine protease, and thrombin. Based on their primary sequences, they share 12 cysteine residues at conserved positions, the catalytic triad residues (His-57, Asp-102, and Ser-195), and the secondary sites Asp-189 and Gly-216 residues [3].

*Bothrops* (*sensu lato*) snakes are widely distributed throughout Central and South American [4], and they are responsible for most venomous snakebites in the Americas and Caribbean [5]. In Brazil, *Bothrops jararaca* (*B*. *jararaca*) is found in wet areas of the southeastern region, while *Bothrops erythromelas* (*B*. *erythromelas*) inhabits xeric and semiarid thorn forest, dry tropical deciduous forest, and open rocky areas, mainly in the northeastern region [4]. *Bothrops* snake venoms usually display high coagulant activity [6], with the genus presenting a well-studied polymorphism. Nahas et al. [7] studied 26 *Bothrops* spp species venoms, and they showed that the *B*. *erythromelas* venom was the only one that could not clot fibrinogen directly, i.e., it was devoid of thrombin-like activity, despite having plasma coagulant activity. Furtado et al. [8] studied the toxicity and biological activities of venoms from the mother and offspring of seven *Bothrops* species, while *B*. *jararaca* showed thrombin-like activity in the venoms from the mother and offspring [8, 9], *B*. *erythromelas* venoms had potent coagulation activity on plasma, but lacked direct fibrinogen coagulant activity. Accordingly, *B*. *erythromelas* venom was shown to be almost totally devoid of reactive bands to antibodies specific to batroxobin, a SVTLE purified from *Bothrops atrox* venom that shows high identity to other SVTLE purified from *Bothrops* venoms [10]. This result indicates that the *B*. *erythromelas* venom that does not show thrombin-like activity, likely due the absence of SVTLE.

Batroxobin, the first SVTLE gene described, has 8 kbp and is organized into five exons [11]. The catalytic residues (His-41, Asp-86, and Ser-178) are codified in exons 2, 3, and 5, respectively. The gene structure of batroxobin is similar to those of the trypsin/kallikrein family [11]. Deshimaru et al. [12] studied cDNAs encoding *Protobothrops flavoridis* and *Trimeresurus gramineus* SVTLE of venom glands; after analyzing the similarity among the 5’-UTR (untranslated region), signal peptide-coding domain, mature protein-coding, and 3’-UTR; they noticed that the mature protein-coding region is less conserved than the others. In addition, they observed those nucleotide substitutions are not neutral in the mature protein-coding region and tend to cause amino acid changes.

Snake venoms present a variability known since ancient times; this variation can be intraspecific (ontogenetic, populational, and geographic), interspecific, intergenus, and interfamily [13]. This diversity might be a consequence of gene duplication and positive selection of toxins, which could be a response to the coevolution of preys, and this fact has a significant consequence in snakebite treatment [14]. Current approaches, such as HPLC and mass spectrometry, show the significant variation of toxin sequences in a single venom, reflecting an adaptation of snakes in different ecological niches or diets [15]. A review of 132 snake species venom proteomes indicated that 90% of venom composition consists of about ten toxin families with a predominance of PLA_2_ (phospholipase type 2), metalloproteinases, serine proteinases, and 3-FTx (three-fingers toxin). The prevalence of each protein family depends on the snake family, genus, or species, and besides the differences in toxin quantity, there are variations in the toxin sequences [16].

To understand SVTLE polymorphism in *Bothrops* snake venoms, we chose to study the venoms and the genomes of *B*. *jararaca* and *B*. *erythromelas* specimens. On that account, we analyzed individual samples of *B*. *jararaca* and *B*. *erythromelas* venoms using coagulation tests in plasma and purified fibrinogen, and evaluated SVTLE genes by a PCR amplification approach using primers designed based on conserved regions of *B*. *atrox*, *B*. *jararaca*, and *B*. *insularis*. This approach aimed to associate the functional SVTLE-like genes with the presence of thrombin-like activity in venoms or the lack of encoding genes with the absence of thrombin-like activity. Toxin genes are sequences that evolve after duplication, and their inactivation might be a consequence of this process, rendering this feature an exciting object of study. Our results could help trace a possible history of birth-and-death of SVTLE genes.

## Results

Thirteen *B*. *erythromelas* venoms and seven *B*. *jararaca* venoms were tested for their clotting activity on bovine plasma (total coagulant activity) and fibrinogen (thrombin-like enzyme activity) (Table 1). *B*. *erythromelas* is reported to contain only metalloproteinases that activate factor X and prothrombin in their venoms [7, 8, 17]. The wide range of the minimum coagulant dose (MCD) values showed that *B*. *erythromelas* venoms displayed various degrees of coagulant activity on plasma. It is essential to notice that the higher the MCD, the lower the coagulant activity. However, no sample of *B*. *erythromelas* venom showed direct thrombin-like activity on fibrinogen. Regarding *B*. *jararaca* venom–which is reported to contain factor X, prothrombin activators, and thrombin-like enzymes [9]–, six samples were tested for coagulant activity both on plasma and fibrinogen. All showed high coagulant activity on plasma, except one (Bj-D, Table 1) did not show coagulant activity on fibrinogen. Other *B*. *jararaca* venom samples showed high coagulant activity on fibrinogen, even one that could not be tested in the plasma due to the low amount of venom available (Bj-E, Table 1).

**Table 1:** Coagulant activities of *B*. *jararaca* and *B*. *erythromelas* snake venoms on bovine plasma and fibrinogen, and DNA sequencing analyses.

Identification	DMC plasma	DMC fibrinogen	DNA purification	PCR	Sequencing
Be 0501–09	404	Absent	Yes	NA	
Be 0501–10	126	Absent	Yes	NA	
Be 0903	857	Absent	Yes	2.8 kbp	Yes*
Be 0904	1695	Absent	Yes	NA	
Be 1101	PI	Absent	Yes	NA	
Be 1104	268	Absent	Yes	NA	
Be 1109	40	Absent	Yes	NA	
RG 577	13	Absent	Yes	NA	
RG 578	56	Absent	Yes	NA	
RG 581	34	Absent	Yes	NA	
RG 633	27	Absent	Yes	NA	
RG 634	113	Absent	Yes	NA	
RG 635	83	Absent	Yes	NA	
Bj-D	729	Absent	Yes	8 kbp	Yes*
Bj-E	NT	275	No		
Bj-H	22	17	Yes	8 kbp	
Bj-G	24	10	No		
Bj-I	33	19	Yes	8 kbp	Yes*
Bj-1	31	26	No		
Bj-2	80	40	Yes	4–5 kbp	

Be and RG identifies *Bothrops erythromelas* and Bj, *B*. *jararaca* snakes. Minimum Coagulation Dose in mg/L. NT if no tested. PI: the coagulating activity is present but is higher than 1600 mg/L. PCR: the size of the amplicon is reported, and NA means that no amplification occurred.

**Fig 1:**
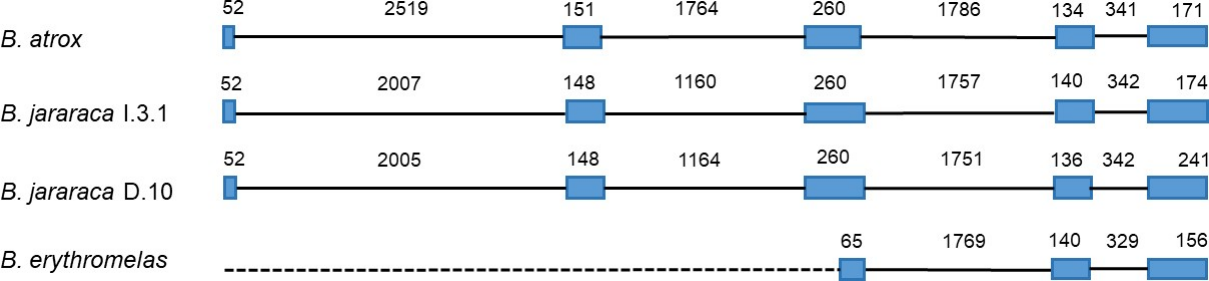
Scheme of SVTLE genes of *Bothrops jararaca* and *B*. *erythromelas*. Numbers inform the sizes of introns (full line) and exons from ATG to stop codon (blue boxes) in nucleotides. The dashed line indicates the deleted region in the *B*. *erythromelas* 0903 gene. GenBank accession numbers: *B*. *atrox* (X12747.1), *B*. *jararaca* I.3.1 (MT547769), *B*. *jararaca* D.10 (MT547770), and *B*. *erythromelas* 0903 (MT536933).

*Gene structures are illustrated in Fig 1 and sequence alignment on S1 Fig.

All DNA samples from *B*. *erythromelas* were studied, but only four samples from *B*. *jararaca* were tested by PCR (Table 1). However, only Bj-I, whose venom displayed thrombin-like activity, and Bj-D, whose venom showed no coagulant activity on fibrinogen, had the SVTLE genes cloned and sequenced (Table 1).

No *B*. *erythromelas* specimen presented amplification using SER-fw and SER-rv primers, except Be 0903, which showed a 2.8 kbp fragment amplification. All DNA tested from *B*. *jararaca* (H, I, and D) presented an amplification of 8 kbp, except Bj-2, with an amplification product of 4–5 kbp. We cloned only the 2.8 kbp fragment from Be 0903 (GenBank MT536933), the 8 kbp from Bj-I.3.1 (MT547769), Bj-D.10, and Bj-D.07 (MT547770 and MT547771) because we had both functional and nonfunctional SVTLE genes. Inserts were sequenced using Sanger’s method, nucleotide sequences were deposited in GenBank, and their alignment is presented in S1 Fig. The gene structures are shown in Fig 1. The *B*. *erythromelas* sequence was 2626 nucleotide (nt) long, contained a partial SVSP gene, presenting a 38 nt long 5’-UTR, complete deletion of the signal peptide in exon 1, intron 1, exon 2, and intron 2, and partial deletion of the 5’-sequence of exon 3.

We observed the conservation of gene structure among SVTLEs (five exons and four introns), similar to the batroxobin gene (X12747.1) described previously [11], despite the size variation. This variability was mainly due to differences in intron sizes, and this variation was also observed in snake β-defensin genes such as crotamine / crotasin [18] and other crotamine-like genes [19]. Our batroxobin-like genes of *B*. *jararaca* I.3.1 (MT547769), D.10 (MT547770) and D.07 (MT547771), and others–e.g., *Crotalus horridus* (GenBank LVCR01039842.1), *C*. *viridis* (PDHV02000010.1), *Protobothrops mucrosquamatus* (GenBank BCNE02033937), and *P*. *flavoridis* (BFFQ01004455.1)–presented the same structure. Table 2 shows the sizes of exons and introns of these genes and the intron phases (phase 0 –intron insertion between codons; phase 1 –intron insertion after the first nucleotide of the codon; phase 2 –intron insertion after the second nucleotide of the codon). The SVTLE gene of *P*. *flavoridis* contained exceptionally long introns 2 and 3 (see Table 2), and it was not used in the alignment (S1 Fig).

**Table 2:** Sizes of exons and introns of snake SVTLE genes, from ATG of the signal peptide to the stop codon in exon 5.

Snake	Exon 1	Intron 1 (phase 1)	Exon 2	Intron 2 (phase 2)	Exon 3	Intron 3 (phase 1)	Exon 4	Intron 4 (phase 0)	Exon 5
*B*. *atrox*	52	2519	151	1764	260	1786	134	341	171
*B*. *jararaca* I.3.1	52	2007	148	1160	260	1757	140	342	174
*B*. *jararaca* D.10	52	2005	148	1164	261	1751	133	342	241
*C*. *viridis*	52	1082	151	1684	260	1776	146	342	183
*C*. *horridus*	52	4126	148	1141	260	1768	140	341	174
*P*. *mucrosquamatus*	52	4191	148	1163	260	1841	140	343	174
*P*. *flavoridis*	52	3671	157	37781	272	22244	140	343	216

The sizes of exons and introns are informed in nucleotides, and the phases of introns are indicated in parentheses. The intron 1 of *Crotalus horridus* has 958 undefined nucleotides (Ns), and the *Protobothrops flavoridis* introns 2 and 3 have unnumbered Ns. GenBank accession numbers: *B*. *atrox* (X12747.1), *B*. *jararaca* I.3.1 (MT547769), *B*. *jararaca* D.10 (MT547770), *C*. *viridis* (PDHV02000010.1), *C*. *horridus* (LVCR01039842.1), *P*. *mucrosquamatus* (BCNE02033937), and *P*. *flavoridis* (BFFQ01004455.1).

Table 3 shows the sequence similarity of SVTLE genes concerning the batroxobin gene. We observed conservation of the signal peptide region, exon 1, which is usual to toxin genes. The similarity of introns is low due to the gaps created by alignment, but the similarity of exons that codify mature SVTLE is high intra-genus. Interestingly, intron 4 is the most conserved intron among SVTLE genes.

**Table 3:** The similarity of SVTLE genes in percent identity, related to the batroxobin gene.

Snake	Exon 1	Intron 1	Exon 2	Intron 2	Exon 3	Intron 3	Exon 4	Intron 4	Exon 5
*B*. *atrox*									
*B*. *jararaca* I.3.1	100	55.3	94	60.7	90	89.5	90	95.6	92
*C*. *viridis*	94.2	21.1	84.8	67.6	80.4	82.6	77.4	92.4	86.8
*C*. *horridus*	94.2	26.8	82.1	56	75.4	82.6	78.6	90.4	82.8
*P*. *mucrosquamatus*	94.2	45.8	81.5	57.7	79.2	80.2	80	91.5	85.1
*P*. *flavoridis*	90.4	44.2	75.6		78.8		79.3	91.8	85.6

*Protobothrops flavoridis* introns 2 and 3 showed many undefined nucleotides, Ns, so they were not used for analysis. Similarity from ATG of the signal peptide to the stop codon of exon 5. GenBank accession numbers: *B*. *atrox* (X12747.1), *B*. *jararaca* I.3.1 (MT547769), *Crotalus viridis* (PDHV02000010.1), *C*. *horridus* (LVCR01039842.1), *P*. *mucrosquamatus* (BCNE02033937), and *P*. *flavoridis* (BFFQ01004455.1).

**Fig 2:**
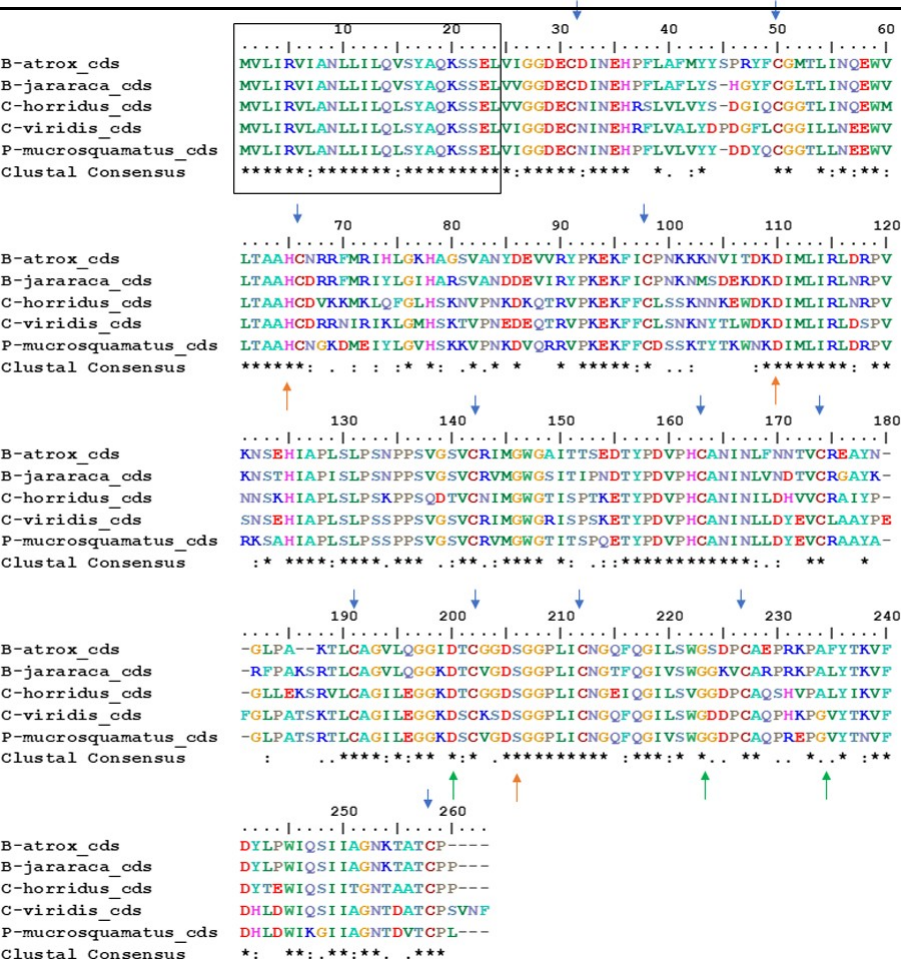
Alignment of amino acids of genomic sequences of SVTLEs analyzed in Table 2. The pseudogenes of *Bothrops jararaca* and *Protobothrops flavoridis* were not analyzed. The black box indicates the signal peptide, blue arrows show the cysteines, orange arrows show the catalytic amino acid triad, and green arrows show the binding pocket.

The coding sequences (cds) of genes described in this work (S1 Fig) were translated, aligned using MUSCLE (EMBL-EBI), and presented in Fig 2. The *P*. *flavoridis* coding sequence was not used because it showed several stop codons in the sequence. The alignment showed in Fig 2 pointed out the presence of: (1) a conserved signal peptide (black box); (2) the catalytic triad (His-Asp-Ser, orange arrows), essential to the thrombin-like activity; (3) conserved cysteines (blue arrows), necessary to the tridimensional structure of the toxin; and (4) the binding pocket (Asp-Gly-Gly, green arrows), vital for substrate specificity. The sequence of *B*. *jararaca* I.3.1 shows Ala234Gly, this substitution also occurred in the SVTLEs of *B*. *atrox* and *C*. *horridus*, and since both are similar amino acids, this may not interfere with substrate specificity.

Batroxobin coding sequence (cds) was used for fishing new sequences in Nucleotide BLAST at NCBI, and only those without premature stop codons were analyzed. Thirty-five SVTLE genes (S1 Table) were aligned and back-translated without stop codons, resulting in 297 sites. They were analyzed with the free software Datamonkey [20], using the fixed effects likelihood method (FEL), which uses a maximum-likelihood approach to infer nonsynonymous (dN) and synonymous (dS) substitution rates. The pervasive positive/diversifying selection evidence was found at 16 sites, and pervasive negative/purifying selection at seven sites, with a p-value threshold < 0.05. The main results are shown in Table 4. Omega is the ratio between dN and dS, and when ω >1 indicates accelerated evolution, while ω < 1 indicates purifying selection. All sites corresponding to Cys, binding pocket, and the catalytic triad did not present suitable statistics, which could be a consequence of ω = 0 or ω = NaN (Not a Number). NaN occurs if all values of dN-dS are on the same side of 0, then all sites are either positive (dN—dS>0) or negative (dN—dS<0). These ω values could indicate the high conservation of sites important to thrombin-like activity. Most sites with pervasive diversifying selection are in exon 2 and the carboxy terminus of the toxin.

**Table 4:** Codons affected by selective pressure in SVTLE genes.

Site	ω	p-value	Site	ω	p-value	
2	>1	0.031	63	NaN	1.000	Cys*
52	>1	0.044	84	NaN	1.000	Cys*
53	>1	0.017	99	NaN	1.000	His^#^
54	>1	0.019	100	NaN	1.000	Cys*
56	>1	0.014	132	0	0.432	Cys*
61	>1	0.040	144	NaN	1.000	Asp^#^
65	>1	0.005	176	NaN	1.000	Cys*
76	<1	0.046	197	NaN	1.000	Cys*
93	<1	0.030	208	NaN	1.000	Cys*
130	>1	0.007	225	NaN	1.000	Cys*
131	>1	0.015	234	0	0.212	Asp^%^
141	<1	0.031	240	0	0.491	Ser^#^
143	>1	0.008	246	NaN	1.000	Cys*
153	>1	0.020	257	NaN	1.000	Gly^%^
198	>1	0.031	261	NaN	1.000	Cys*
222	>1	0.041	269	2.15	0.585	Gly^%^
237	<1	0.040	292	NaN	1.000	Cys*
249	<1	0.013				
263	<1	0.010				
281	<1	0.041				
290	>1	0.040				
291	>1	0.046				
294	>1	0.022				

The sequences were analyzed in Datamonkey, analysis **PMID 15703242**: ω >1 indicates accelerated evolution while ω< 1 indicates purifying selection, ω = NaN (Not a Number). Amino acids essential to the tridimensional structure of the toxin (Cys)*,

the coagulant activity (His-Asp-Ser)^#^,

and substrate specificity (Asp-Gly-Gly)^%^.

**Fig 3:**
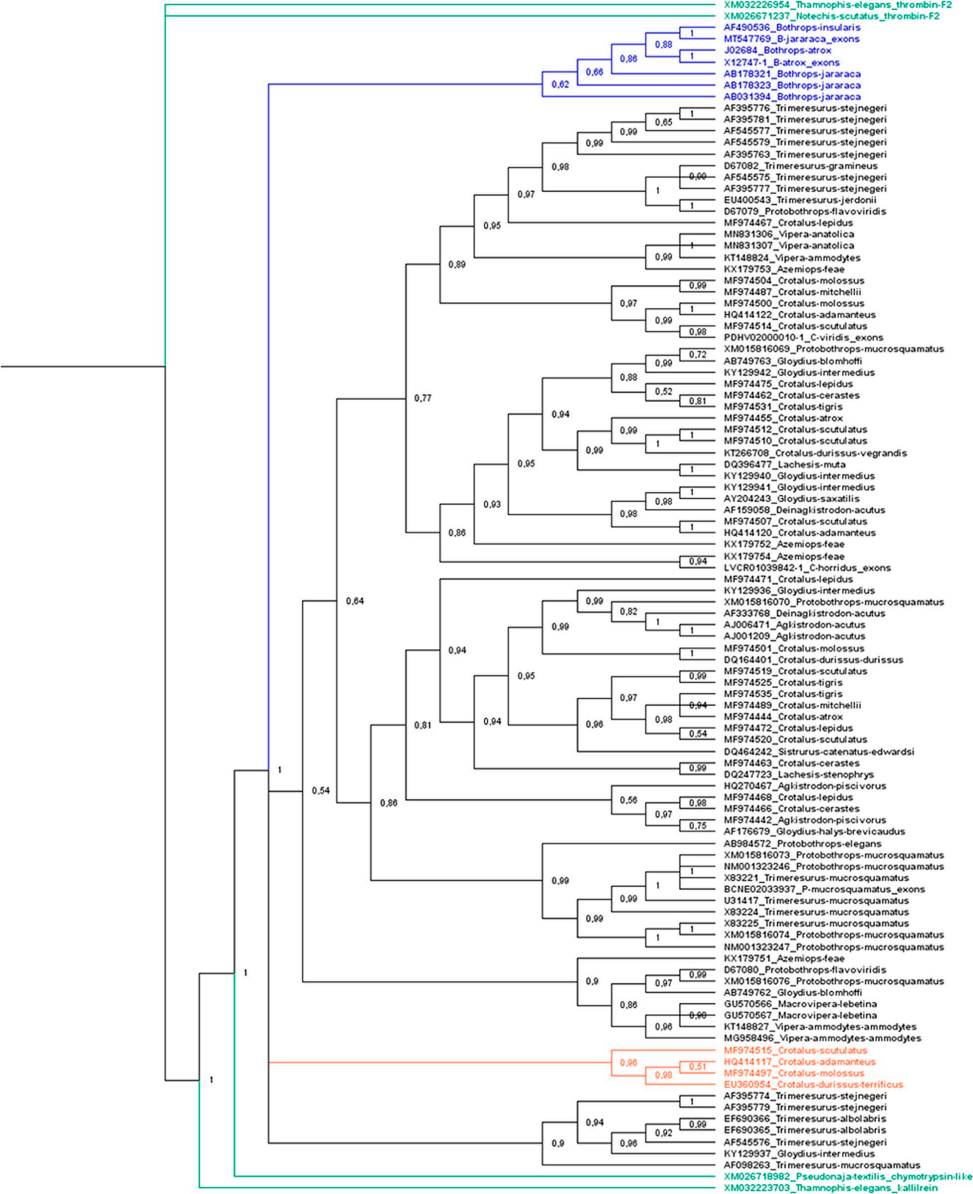
Cladogram of SVTLE coding sequences. The tree was constructed with Mr. Bayes 3.2.6 using *Thamnophis elegans* thrombin (XM032226954) as the outgroup and GTR as the nucleotide substitution model. In green, thrombin, chymotrypsin, and kallikrein codifying sequences; in blue, the batroxobin-like sequences; and orange, the gyroxin-like sequence.

We utilized the cds in phylogenetic reconstruction taking into consideration two facts: (1) the variation in intron size is strongly pronounced, thus this characteristic only increases the matrix size, and it is not informative; (2) Oliveira et al. [21] used only cds of β-defensin genes, short sequences with 2 kbp approximately, and obtained a good relationship and resolution in the phylogenetic tree. The phylogenetic analysis was done using the SVTLE cds, including cds of snake chymotrypsin, thrombin, and kallikrein. The tree (Fig 3) was constructed using Mr. Bayes 3.2.6 [22], using *Thamnophis elegans* thrombin as the outgroup (XM032226954) and the GTR nucleotide substitution model. The tree illustrates that SVTLE sequences had a closer relationship to chymotrypsin/kallikrein than to thrombin, as observed by Itoh et al. [11] when describing the batroxobin gene. SVTLE genes were organized into four groups: a batroxobin-like group (*Bothrops* sequences), a large group including the majority of SVTLE sequences from Viperidae snakes, a gyroxin-like group [23] (*Crotalus* sequences), and, finally, a group containing *Trimeresurus*/*Gloydius-*related sequences. The large group comprises all other diverse SVTLE sequences of the Viperidae family.

## Discussion

Snake venoms are a complex mixture of proteins with many biological activities [24] and present variabilities at different levels; in this work, we observed an intraspecific polymorphism in *B*. *erythromelas* and *B*. *jararaca* venoms. Regarding *B*. *erythromelas* snake venom, geographic variation was noticed for plasma coagulant activity, so that venoms from individuals inhabiting Rio Grande do Norte state (RN, Brazil) and Bahia state (BA, Brazil) showed lower coagulant activity than those from Pernambuco state (PE, Brazil). On the other hand, *B*. *jararaca* venoms showed reduced MCD variation for plasma coagulant activity due to the proximity of capture locality. Toxinologists usually believe that thrombin-like activity is an interspecific variation and that *B*. *jararaca* venom always clots fibrinogen. However, we found a specimen of *B*. *jararaca* whose venom was unable to clot fibrinogen. This finding indicates that this venom characteristic is intraspecific and not interspecific, as considered previously. The lack of thrombin-like activity in *B*. *jararaca* venom had been indirectly observed previously by others (Serrano S.M.T., Instituto Butantan). Polymorphism for thrombin-like activity in snake venoms and other venom activities has significant consequences for the treatment of snakebites, once variation in the clinical picture may therefore be observed [14].

Jorge et al. [25] detected serine proteases in the venoms of five populations of *B*. *erythromelas*, but we did not find thrombin-like activity in any of the venoms tested. Serrano [26] described in a review some SVTLE with weak clotting activity on fibrinogen, e.g., thrombocytin [27, 28], or utterly devoid of fibrinogen-clotting activity, e.g., PA-Bj [29]. An exception was observed by Vasconcelos [30], who observed *B*. *erythromelas* venoms from specimens captured on the island of Itaparica, Bahia State, which clotted fibrinogen. Interestingly, those are individuals from an isolated population, and according to Jorge et al. [25] analysis, this was the population with the highest proportion of serine proteinases in venoms. In our study, the individual venoms from snakes inhabiting the continental regions of the northeastern states did not have thrombin-like activity.

Some venom polymorphism studies have shown that genetic factors are responsible for controlling the expression of certain toxins in snake venoms. For example, a sequence of about 411 bp in the upstream region of the toxic IA group PLA_2_ genes of *Laticauda semisfaciata* is determinant for its high expression; its insertion may have been responsible for PLA_2_ expression in the venom gland [31]. On the one hand, the lack of the crotamine gene is responsible for the lack of crotamine activity in the *Crotalus durissus terrificus* venom [18]. Another gene deletion was also described to a different toxin, the PLA_2_, in rattlesnakes [32]. On the other hand, the increase in the number of functional genes may increase the proportion of crotamine in the venom [33]. Moreover, we cannot forget other gene regulation mechanisms, which imply the gene presence and differential expression according to the ontogenetic development. Santoro et al. [10] studied six hybrid snakes from the crossing of *B*. *erythromelas* (mother) with *B*. *neuwiedi* (father). They showed that in the F1 breed, only one hybrid offspring presented thrombin-like activity from six months onwards, while all the analyzed offsprings venoms were able to coagulate fibrinogen from 24 months onwards. Moreover, Shibata et al. [34], studying the venom gland’s transcriptome and the genome of pit viper Habu snake, *Protobothrops flavoviridis*, observed that some toxin genes were low represented in the genome compared to the gland venom transcriptome and concluded that the richness of cds could be a consequence of alternative splicing.

Gene families may undergo expansion or contraction and may even be lost. There are several examples of gene amplification, but evidence of gene losses remains rare and, in most cases, results from neutral processes [35]. In virtue of the lack of gene sequences, it is challenging to infer losses, but comparative studies can be carried out using two related species [35]. In this sense, we illustrate evidence of gene loss in the *Bothrops* genus, where one individual *B*. *jararaca* (Bj-D) did not have thrombin-like activity in its venom. Moreover, its genome contained a gene with a complete SVTLE structure but mutations that rendered the enzyme inactive. Moreover, we found examples of *B*. *erythromelas* individuals without thrombin-like activity with incomplete gene or lack of genes. Such findings illustrate that the lack of thrombin-like activity results from diverse causes, as nonsense or frameshift mutations in *B*. *jararaca* and gene loss in *B*. *erythromelas*.

SVTLE genes are part of a multigene family that comprises genes that descend from a common ancestor and share similar biological functions and genomic sequences. Gene duplication, followed by nucleotide substitution and positive selection, can explain the various activities of serine proteases and other toxins present in snake venoms, as well as the existence of pseudogenes [36]. The gene birth-and-death model promotes genetic diversification; in this model, the copies may diverge in sequence until they do not have large regions of similarity, creating new gene families; some copies may degenerate to pseudogenes or be deleted by unequal crossing-over [37]. Our data showed that the SVTLE genes underwent accelerated evolution, and we suppose that the high rate of nucleotide substitution could have caused gene inactivation, leading to an accumulation of deletions. The analysis of 109 processed mouse and rat pseudogenes demonstrated that deletions are most frequent (occurring once every 40 nt substitutions) than insertions (occurring once every 100 nt substitutions) on average, and that this frequency increases with time [38]. Furthermore, loss of the pseudogene can occur due to accelerated genetic drift for reasons that are not readily apparent [39].

We have achieved a few sequences with our approach. It points out our strategy’s bottleneck, since it depends on sequences complementary to the primers used for PCR, the efficiency of the amplification of large sequences (about 7 kbp), and the cloning and amplification of these large inserts in bacterial plasmids. However, despite the PCR approach difficulty, the nucleotide analysis of gene families, including their pseudogenes, provided us with a powerful phylogenetic study tool. Investigating the genome evolution among different species and populations within one species, focusing on pseudogenes could significantly impact gene evolution comprehension [39].

We associated the lack of thrombin-like activity to the presence of inactive genes in *B*. *jararaca* Bj-D, while in *B*. *erythromelas* to the presence of a partial gene, in Be 0903, and/or the lack of genes or sequences complementary to the primers used for PCR amplification in other individuals. We infer that the SVTLE gene, functional in *B*. *jararaca* species, was inactivated by nonsense and frameshift mutations. Similarly, Li et al. [40] showed that in *Aisysurus eydouxii* the inactivation of the three-finger toxin gene was due to a deletion of a dinucleotide, resulting in frameshift or premature stop codon. This inactivation may have occurred in an ancestor of *B*. *erythromelas*, and sequence deletion or crossing-over events have been taking place over time. Since venoms are complex mixtures of toxins, they do not depend on a unique biological activity for immobilizing or eating preys. It creates a neutral selection environment that allows the loss of a nonfunctional gene by genetic drift. Despite the most common distribution in Crotalinae snakes, our search for SVTLE coding sequences showed that they are also present in venom glands of snakes from the Elapidae and Colubridae families, in addition to the subfamilies Viperinae and Azemiopinae. To better understand the SVTLE gene family evolution, it would be interesting to investigate gene sequences in the population of *B*. *erythromelas* inhabiting the Island of Itaparica, whose venom showed thrombin-like activity [30], thus completing the panorama of this family.

## Materials and methods

### Snakes

Table 5 lists the features of individuals whose venom and blood samples were used herein. The experimental procedures involving snakes followed Brazilian Guidelines (the Institutional Animal Care and Use Committee from Butantan Institute—CEUAIB 8094050216).

**Table 5:** Snakes whose venoms have been tested for coagulant activity.

Species	Identification	Sex	Locality
*B*. *erythromelas*^1^	Be 0501–09	Female	Natal-RN
*B*. *erythromelas*^1^	Be 0501–10	Male	Natal-RN
*B*. *erythromelas*^1^	Be 0901	Male	Ibitira-BA
*B*. *erythromelas*^1^	Be 0903	Female	Ibitira-BA
*B*. *erythromelas*^1^	Be 0904	Female	Ibitira-BA
*B*. *erythromelas*^1^	Be 1101	Female	Ibitira-BA
*B*. *erythromelas*^1^	Be 1104	Male	Ibitira-BA
*B*. *erythromelas*^1^	Be 1109	Female	Ibitira-BA
*B*. *erythromelas*^2^	RG 577	Female	Petrolina-PE
*B*. *erythromelas*^2^	RG 578	Female	Petrolina-PE
*B*. *erythromelas*^2^	RG 581	Female	Petrolina-PE
*B*. *erythromelas*^2^	RG 633	Female	Petrolina-PE
*B*. *erythromelas*^2^	RG 634	Female	Petrolina-PE
*B*. *erythromelas*^2^	RG 635	Female	Petrolina-PE
*B*. *jararaca*^3^	Bj D^4^	Male	São Roque—SP
*B*. *jararaca*^3^	Bj E	Female	Santana de Parnaíba—SP
*B*. *jararaca*^3^	Bj H	Male	Embu das Artes—SP
*B*. *jararaca*^3^	Bj G	Female	Itapecerica da Serra—SP
*B*. *jararaca*^3^	Bj I^4^	Male	Ibiuna—SP
*B*. *jararaca*^3^	Bj 1	Female	Araçariguama—SP
*B*. *jararaca*^3^	Bj 2	Male	São Roque—SP

^1^Snakes from Laboratory of Herpetology of the Butantan Institute.

^2^Snakes from Laboratory of Poisonous Animals and Toxins—LAPTx of Pernambuco Federal University.

^3^Samples from project approved in CEUAIB 1349/14.

^4^Voucher: Bj-D, IBSP 88.139, and Bj-I, IBSP 89.192. States in Brazil where the snakes were captured: RN (Rio Grande do Norte), BA (Bahia), PE (Pernambuco), SP (São Paulo).

### Venoms

Venoms were collected manually and maintained in ice. After centrifugation at 5000 *g* for 10 min at 4°C, the supernatants were stored at –20°C until lyophilization. The lyophilized venom was stored at -20°C and dissolved immediately before the coagulation tests.

#### Coagulation studies

A modification of the minimum coagulant dose (MCD) [41] was used to compare the coagulant activity of different venom samples on two substrates: citrated bovine plasma (MCD-P) (CEUA/FMVZ 1726060917) and bovine fibrinogen (MCD-F) solution [9, 42]. MCD was defined as the least amount of venom that clotted plasma or fibrinogen solution in 60 s at 37°C. Citrated bovine plasma samples were used to evaluate both the procoagulant (factor II and X activators) and the thrombin-like activities of *Bothrops* venoms, while the fibrinogen solution served only to determine the activity of thrombin-like enzymes [9]. Briefly, bovine fibrinogen (Sigma F8630) was dissolved at a final concentration of 2 mg/mL clottable protein, and lyophilized venom samples were dissolved at a final concentration of 8 mg/mL, both in 154 mM NaCl solution. Venom samples and the fibrinogen were freshly diluted before clotting assays. Venom samples were two-fold serially diluted in 154 mM NaCl and maintained in an ice bath during assays. Aliquots of 25 μL of venom were added to 100 μL of bovine plasma or fibrinogen, and the clotting time was recorded in a Start4 coagulometer (Stago, France). Tests were carried out in triplicate, using final venom concentrations ranging from 0.4 μg/mL to 1.6 mg/mL. Regression analyses of final venom concentration plots against clotting time were performed for each venom sample on two substrates, using the software CurveExpert version 1.40, which calculated the best fit and MCD values.

### DNA purification from blood and tissue samples

Blood samples were taken from the caudal vein using 15 mM sodium citrate (final concentration) as an anticoagulant. Samples from *B*. *erythromelas* and *B*. *jararaca* specimens from the Laboratory of Herpetology were stored at -20°C until purification using ZR Genomic DNA–tissue miniprep. *B*. *erythromelas* blood from the Pernambuco state was dropped in FTA elute Microcard (Thermo-Fischer Scientific) to preserve the DNA during shipment. Briefly, a 3 mm diameter sample was punched out using a Harris Uni-Core Punch 3.0 mm (Thermo-Fischer Scientific). The disc was rinsed with 0.5 mL of ultrapure water and vortexed five times; then, the disc was transferred to a new microtube. The DNA was purified from the disc using 50 μL of ultrapure water, followed by heating to 95°C for 30 min and vortexing for 1 min. After centrifugation, the disc was withdrawn.

**PCR and cloning.** Primers:

SER-fw: TAAGTAAGGGACTGGGATCT;
SER-rv: GGATATGGTTAGGGATATAGAAGAG.

For *B*. *jararaca*: in 25 μL of reaction, it was used 0.4 μM of each primer, 100 ng of DNA sample, 250 μM of dNTPs, and 0.5 μL of PfuUltra II Fusion HS DNA polymerase (Agilent Technologies). The amplification process consisted of 95°C—2 min; followed by 30 cycles of 95°C—30 s, 55°C—30 s, 72°C—2 min; finally, 72°C—3 min.

For *B*. *erythromelas*: in 25 μL of reaction, it was used 0.4 μM of each primer, 200 ng of DNA sample, 250 μM of dNTPs, and 0.5 μL of PfuUltra II Fusion HS DNA polymerase (Agilent Technologies). The amplification process consisted of 92°C—2 min; followed by 30 cycles of 92°C—30 s, 55°C—30 s, 68°C—5 min; finally, 68°C—5 min.

The amplified sequences were purified from agarose gel using the Zymoclean Gel DNA Recovery Kit (ZymoResearch) and cloned into pSC-B-amp/kan vector using the StrataClone Blunt PCR Cloning kit (Stratagene). The transformants were selected after plating on LB with 100 μg/mL ampicillin. Before the plating, it was spread 10 μL of 0.4 M Isopropyl β-D-thiogalactoside **(**IPTG) and 20 μL of 4% 5-Bromo-4-chloro-3-indolyl β-D-galactopyranoside (X-Gal). Isolated bacterial white colonies were cultured in LB medium with 100 μg/mL of ampicillin, and the plasmids were purified using Zyppy Plasmid Miniprep Kit (ZymoResearch). This work was developed in a biosecurity laboratory (CIBIO AREA-32, CQB 39/98 –Instituto Butantan).

#### Sequencing

Aliquots of 300–350 ng of plasmid DNA and 1.6 pmol of primer were used to sequence using the Sanger method (BigDye® Direct Cycle Sequencing Thermo Fisher Scientific) at the Laboratory of Bacteriology of Instituto Butantan. Primers used are described in S2 Table.

#### Sequence analysis

Low quality and vector sequences were removed using SeqScanner (Applied Biosystems) and VecScreen (NCBI), respectively. Sequences were edited visually using BioEdit v7.1.3 software [43]. The genes were assembled using Geneious R.6.0.6 [44] and aligned using MAFFT [45]. The evolutionary codon rate was estimated in DataMonkey (http://www.datamonkey.org, [20]). The phylogenetic tree was constructed using Mr. Bayes 3.2.6 [22], thrombin-F2 as outgroup, and the following parameters: lset applyto = (all) nst = 6 rates = invgamma; prset applyto = (all) aamodelpr = mixed; unlink revmat = (all) shape = (all) pinvar = (all) statefreq = (all) tratio = (all); showmodel; mcmc ngen = 10000000 printfreq = 1000 samplefreq = 100 nchains = 4 temp = 0.2 checkfreq = 50000 diagnfreq = 1000 stopval = 0.01 stoprule = yes; sumt relburnin = yes burninfrac = 0.25 contype = halfcompat; sump relburnin = yes burninfrac = 0.25; log stop; end. The tree was edited using FigTree v. 1.4.4 (by Andrew Rambaut, Institute of Evolutionary Biology, University of Edinburgh).

### Ethics statement

This study was carried out according to the National Council for Control of Animal Experimentation (CONCEA). The Ethics Committee approved the protocol on Animal Use of the Butantan Institute (CEUAIB), protocol number 8094050216. The blood and venom collection were made thinking to minimize stress and suffering.

## Supporting information

S1 Fig SVTLE genes alignment. Sequences of *Bothrops atrox* (X12747.1), *Crotalus horridus* (LVCR01039842.1), *C*. *viridis* (PDHV02000010.1), *Protobothrops mucrosquamatus* (BCNE02033937), *B*. *jararaca* I (MT547769), and *B*. *jararaca* D (MT547770- D.10 and MT547771-D.07) were aligned from ATG of exon 1 to stop codon of exon5. CLUSTAL multiple sequence alignment by Mafft [45].
(DOCX)

S1 Table Sequences used in selection analysis. Sequences used in Datamonkey analysis, only the coding sequences without stop codons were analyzed.
(DOCX)

S2 Table Primers using in Sanger sequencing. M13 primers are complementary to borders of plasmid vector. BE primers are complementary to *Bothrops erythromelas* sequences. BJ primers are complementary to *Bothrops jararaca* sequences.
(DOCX)
