# Supplementary material for: The absence of thrombin-like activity in Bothrops erythromelas venom is due to the deletion of the snake venom thrombin-like enzyme gene
Source: PLoS One. 2021 Apr 27;16(4):e0248901. doi: 10.1371/journal.pone.0248901 (PMC8078745; doi:10.1371/journal.pone.0248901)
Supplement: S1 Fig — Sequences of Bothrops atrox (X12747.1), Crotalus horridus (LVCR01039842.1), C. viridis (PDHV02000010.1), Protobothrops mucrosquamatus (BCNE02033937), B. jararaca I (MT547769), and B. jararaca D (MT547770- D.10 and MT547771-D.07) were aligned from ATG of exon 1 to stop codon of exon5. CLUSTAL multiple sequence alignment by Mafft [45]. (DOCX) [file pone.0248901.s001.docx]

CLUSTAL multiple sequence alignment by MUSCLE (3.8)

Exon 1 Intron 1

B-atrox ATGGTGCTGATCAGAGTGATAGCAAACCTTCTGATATTACAGGTTTCTTACGgtaagagc

C-horridus ATGGTGCTGATCAGAGTGCTAGCAAACCTTCTGATACTACAGCTTTCTTACGgtaagagc

C-viridis ATGGTGCTGATCAGAGTGCTAGCAAACCTTCTGATACTACAGCTTTCTTACGgtaagagt

P-mucrosquamatus ATGGTGCTGATCAGAGTGCTAGCAAACCTTCTGATACTACAGCTTTCTTACGgtaagagc

B-jararaca_I.3.1 ATGGTGCTGATCAGAGTGATAGCAAACCTTCTGATATTACAGGTTTCTTACGgtaagaac

B-jararaca_D.10 ATGGTGCTGATCAGAGTGATAGCAAACCTTCTGATATTACAGGTTTCTTACGgtaagaac

B-jararaca_D.07 ATGGTGCTGATCAGAGTGATAGCAAACCTTCTGATATTACAGGTTTCTTACGgtaagaac

****************** ***************** ***** ***************

B-atrox caggacaatggagcaaagggagaacagagctttaggtgtctttttcggtttaaacctcca

C-horridus ctggac---ggagcaaagggtggacggagctttaggtgtctttttcggtttaaacctcac

C-viridis ctggacaatggagcaaagggtggacggagctttaggtgtctttttctgtttaaacctcca

P-mucrosquamatus ctggacaatggagcaaggggtggacggagctttaggtgtctttttcggtttaaacctcca

B-jararaca_I.3.1 caggacaatggagccaagggtgaacagagctttaggtgtctttttcagtttaaacctcca

B-jararaca_D.10 caggacaatggagccaagggtgaacagagctttaggtgtctttttcagtttaaacctcca

B-jararaca_D.07 caggacaatggagccaagggtgaacagagctttaggtgtctttttcagtttaaacctcca

* **** ***** * *** * ** ******************** ***********

B-atrox gttacaggagacaacttgcatgtaattattaactgtagatgtacaagcaggacacaagta

C-horridus gttacaggaataaacttgcatgtaattattaactgtagatgtacaagcaggtcacaagta

C-viridis gttacaagagtcaacttacatgtaattattaactatagatgtgtaagcaggtcacaagta

P-mucrosquamatus ggtacaggaataaacttgcatgtaattattaactgtagatgtacaagcaggtcacaagta

B-jararaca_I.3.1 gttacaggagtaaacttgcatgtgattattaactatagatgtacaagcaggtcacaagta

B-jararaca_D.10 gttacaggagtaaacttgcatgtgattattaactatagatgtacaagcaggtcacaagta

B-jararaca_D.07 gttacaggagtaaacttgcatgtgattattaactatagatgtacaagcaggtcacaagta

* **** ** ***** ***** ********** ******* ******* ********

B-atrox gagagtgcgcgaacgaggcccagttggtgcaaatggggaagacca-tgctgta-ttgtaa

C-horridus gagagtgtgcgaagaagtcccggttggtgcaaatggggaagaccattgctgtatttgtaa

C-viridis gagagtgggtgaagaaggcccagttggtgcaaatggggaagaccattgctgtatttgtaa

P-mucrosquamatus gagagtggatgaagaaggcccggttggtgcaaatggggaagaccattgctgtatttgtaa

B-jararaca_I.3.1 gagagtgtgcgaagaaggcctggacggtgcaaatggggaagaccattgctgtatttgtaa

B-jararaca_D.10 gagagtgtgcaaagaaggcctggacggtgcaaatggggaagaccattgctgtatttgtaa

B-jararaca_D.07 gagagtgtgcaaagaaggcctggacggtgcaaatggggaagaccattgctgtatttgtaa

******* ** ** ** * ******************** ******* ******

B-atrox tgatagtatgaattgatagaattatt-atatcacgaaa-----actagccacgttaaaaa

C-horridus tgatagtatgaattgatacatttattcatatcagcaac-----actagcca-gttaaaaa

C-viridis tgatagtatgaatttatacatgtattcatatcagttaaaaagtactagcca-gttaaaac

P-mucrosquamatus tgatagtatgaattgatacatttattcatagcagcaaa-----actagcca-gttaaaaa

B-jararaca_I.3.1 tgatagtatgaattgatacatttattcatatcagcaaa-----actagcca-gttaaaaa

B-jararaca_D.10 tgatagtatgaattgatacatttattcatatcagcaaa-----actagcca-gttaaaaa

B-jararaca_D.07 tgatagtatgaattgatacatttattcatatcagcaaa-----actagcca-gttaaaaa

************** *** * **** *** ** * ******** *******

B-atrox tatattaatcccaaatatggttagctttcttttgtgtttctattta-ttggcaactgact

C-horridus tata-taatcccaaatatggtcagcttccttttgtgtttctatttatttggcaactgact

C-viridis tata-taatcccaaatatggtcagcttcctttcgtgtttctatttatttggcaactgact

P-mucrosquamatus tata-taatcccaaatatggtcagcttcctttcgtgtttctatttatttggcaactgact

B-jararaca_I.3.1 tatg-tagtcccaaatatggtcagcttcctttcctgtttctatttatttgacaactgact

B-jararaca_D.10 tatg-tagtcccaaatatggtcagcttcctttcctgtttctatttatttgacaactgact

B-jararaca_D.07 tatg-tagtcccaaatatggtcagcttcctttcctgtttctatttatttgacaactgact

*** ** ************* ***** **** ************ *** *********

B-atrox tcaatagagaaagataacttattaattaatataagatgcattgagaaaaaagttggggag

C-horridus ttaatagaggaagataacttattaattaatataagatgcatttagaaaaaagttggggag

C-viridis tcaatagaggaagataacttattaagtaatataaaatgcattgagaaaaaagttgggaag

P-mucrosquamatus tcaatagaggaagataacttattaattaatataagatgcaatgagaaaaaagttggggag

B-jararaca_I.3.1 tcaatagaggaagataacttattaattaatataagatgcattgagaaaaaagttggagag

B-jararaca_D.10 tcaatagaggaagataacttattaattaatataagatgcattgagaaaaaagttggagag

B-jararaca_D.07 tcaatagaggaagataacttattaattaatataagatgcattgagaaaaaagttggagag

* ******* *************** ******** ***** * ************* **

B-atrox gagtttttagtgttaaggtggtcttt-aaaaaatggtgtatgagatggccattgattttt

C-horridus gggttttcagtgttaaggtagtctttaaaaaaatggtgtatgagatggccattgattttt

C-viridis gggttttcagtgttaaggtggtcttt-aaaaaatggtgtatgagatggccattgattttt

P-mucrosquamatus gggttttcagtgctaaggtggtcttt-aaaaaatggtatatgagatggccattgattttt

B-jararaca_I.3.1 gggtttttagtgttaaggtggtcttt-aaaaaatggtgtatgagatggccattgatgttt

B-jararaca_D.10 gggtttttagtgttaaggtggtcttt-aaaaaatggtgtatgagatggccattgatgttt

B-jararaca_D.07 gggtttttagtgttaaggtggtcttt-aaaaaatggtgtatgagatggccattgatgttt

* ***** **** ****** ****** ********** ****************** ***

B-atrox gtccaaactggtaaaataaatctgttgtatttaacctagctccccttctgttatatttgc

C-horridus gtccaaaatggtaaaataaatctgttgcatttaacctagctcccattctcttagatttgc

C-viridis gtccaaactggtaaaataaatctgttggatttaacctagctcccattctattagatttgc

P-mucrosquamatus gtccaaactggtaaaataaatctgttggatttaacctagctcccattctgttagatctgc

B-jararaca_I.3.1 gtccaaagtggtaaaataaatctgttggatttaacctagttcccattctgttagctttgc

B-jararaca_D.10 gtccaaagtggtaaaataaatctgttggatttaacctagttcccattctgttagctttgc

B-jararaca_D.07 gtccaaagtggtaaaataaatctgttggatttaacctagttcccattctgttagctttgc

******* ******************* *********** **** **** *** * ***

B-atrox tgcattatacagggtgcaatatctgggtcattgcttgtgaaataaatatcataa-cggtg

C-horridus tgcattatacagcgtacaatatctgggtcattgcttctgaaacaaatatcaaaaccggta

C-viridis tacatggtacagtatgcaataccttggtcattgcttctgaaacaaatatcaaaaccagta

P-mucrosquamatus tgcattatagagcgtgcaatatctgggtcattgcttctgaaacaaatctcaaaaccggta

B-jararaca_I.3.1 tgcattatacaggatgcaatatctgggtcattgcttctgaaacaaatatcaaaaccggta

B-jararaca_D.10 tgcattatacagggtgcaatatctgggtcattgcttctgaaacaaatatcaaaaccggta

B-jararaca_D.07 tgcattatacagggtgcaatatctgggtcattgcttctgaaacaaatatcaaaaccggta

* *** ** ** * ***** ** *********** ***** **** *** ** * **

B-atrox tgtagggattttactgccatcaatgttggctcaaactggttttagtgaagtaaatatcta

C-horridus tgtagggaatttactgccatcagtgttagctcaaactgtttttagtgaactaaata-cta

C-viridis tgcatggattttactgccatcaatgttggctcaaactgtttttagtgaaccaaata-ata

P-mucrosquamatus tttagggattttactgctatcaatgtgggctcaaactgtttttagtgaactaaata-cta

B-jararaca_I.3.1 tgtagggattttactgccatcaatgttggctaaaactatttttagtgaactaaata-cta

B-jararaca_D.10 tgtagggattttactgccatcaatgttggctaaaactatttttagtgaactaaata-cta

B-jararaca_D.07 tgtagggattttactgccatcaatgttggctaaaactatttttagtgaactaaata-cta

* * *** ******** **** *** *** ***** ********** ***** **

B-atrox atgttatgaacttcccatagattaaattgcattttcatcaatcacttccccactttcact

C-horridus atgttatcaacttcccatagattaaattccattttcatcaatctcttccccactttcact

C-viridis atgttatcaacttcccactggcagacg---------------------------------

P-mucrosquamatus atcttatcaacttcccatagattgaattccatttttatcaatctcttccccagtttcact

B-jararaca_I.3.1 gtgttatcaacttcccatagattaaattccattttcatcaatctcttctccactttcact

B-jararaca_D.10 gtgttatcaacttcccatagattaaattccattttcatcaatctcttctccactttcact

B-jararaca_D.07 gtgttatcaacttcccatagattaaattccattttcatcaatctcttctccactttcact

* **** ********* * *

B-atrox ccttctgccccctattctccccatcacgaggccatgtattttgcttagcaatgcagtaca

C-horridus acttctgccccctattctccccatcacaaggccatatattttgcttaggaatgcagtaca

C-viridis --------------------------------------------ttaaaaatggaattc-

P-mucrosquamatus ccttctcttccctattctccacatcatgaggccatatattttgcttagcaatgcagtaca

B-jararaca_I.3.1 ccttttgtcccctattctccacatcacgaggccatgtattttgcttagcaatgcagtaca

B-jararaca_D.10 ccttttgtcccctattctccacatcacgaggccatgtattttgcttagcaatgcagtaca

B-jararaca_D.07 ccttttgtcccctattctccacatcacgaggccatgtattttgcttagcaatgcagtaca

*** **** * * *

B-atrox gcaagatcaga--ctggaccaaatgcttctaaatgtcccatc-ctttagaagctgatctg

C-horridus gcaatgcaatagcctaagtta---------------------------------------

C-viridis ------------------------------------------------------------

P-mucrosquamatus gcaagatcagactctggaccaaatgcttctaaatgtccatcctttttagaagctcatctg

B-jararaca_I.3.1 gcaagatcagactctggaccaaatgcttctaaatgtccatcctttttggaagctcatctg

B-jararaca_D.10 gcaagatcagactctggaccaaatgcttctaaatgtccatcctttttggaagctcatctg

B-jararaca_D.07 gcaagatcagactctggaccaaatgcttctaaatgtccatcctttttggaagctcatctg

B-atrox aactattcttcttacttcaattaattagcgtaatctagtcggcagtgataccattaagtt

C-horridus ---------------tttatttatttagc-------------------------------

C-viridis ---------------------------------------------tgatgcc--------

P-mucrosquamatus aactattcttctgactttaactgattagcgtaatctgactggcagtgataccattaagtt

B-jararaca_I.3.1 aactattcttcttacttcaactaattagcgtaatctgactggcagtgataccattaaatt

B-jararaca_D.10 aactattcttcttacttcaactaattagcgtaatctgactggcagtgataccattaaatt

B-jararaca_D.07 aactattcttcttacttcaactaattagcgtaatctgactggcagtgataccattaaatt

B-atrox actgagtagccttctgtatttttaacaacaataatagtgtccatatatatcatttttttc

C-horridus ------------------------------------------------------------

C-viridis ------------------------------------------------------------

P-mucrosquamatus actgagcagtcttctatatttataccaacaataatagcgtccatatatagaattc-----

B-jararaca_I.3.1 actg--------------------------------------------------------

B-jararaca_D.10 actg--------------------------------------------------------

B-jararaca_D.07 actg--------------------------------------------------------

B-atrox ttatcagcttctgttattttcataaaaatcatgttattaattcaccatata--ttatttt

C-horridus ------------------------------------------------------------

C-viridis ------------------------------------------------------------

P-mucrosquamatus ----------------ttttcataaaagtcatcttattgattcaccatatatcttatttt

B-jararaca_I.3.1 ------------------------------------------------------------

B-jararaca_D.10 ------------------------------------------------------------

B-jararaca_D.07 ------------------------------------------------------------

B-atrox cataaatcttgtttaagctcaatttatccataatatc--aacaattcttatcaatcataa

C-horridus -------------------------------aatattcaaacaa----------------

C-viridis ------------------------------------------------------------

P-mucrosquamatus cataaatcttgtttaagctcaatttctccataatattcaaacaattcttatcaatcataa

B-jararaca_I.3.1 ---------------agctcaatttctccataatattcaaacaa-tcttatcaattacag

B-jararaca_D.10 ---------------agctcaatttctccataatattcaaacaa-tcttatcaattacag

B-jararaca_D.07 ---------------agctcaatttctccataatattcaaacaa-tcttatcaattacag

B-atrox ttttctcaataattaacatctaaatacttcaaatttccattt-aatatatcatcccacta

C-horridus ------------------------------------------------------------

C-viridis ------------------------------------------------------------

P-mucrosquamatus ttttctcaataattaacatctaaatatccaaattttccacttaaatatatcatcccagta

B-jararaca_I.3.1 ttttcacaataattaacatctaaatatccaatttttccacttaaatat------------

B-jararaca_D.10 ttttcacaataattaacatctaaatatccaaattttccacttaaatat------------

B-jararaca_D.07 ttttcacaataattaacatctaaatatccaaattttccacttaaatat------------

B-atrox ttt--------catatattatacaaatactatgctacttatgtatc-------------a

C-horridus ------------------------------------------------------------

C-viridis ------------------------------------------------------------

P-mucrosquamatus ttttcatattacatatattatagaaatactatacttcttatctatcatcacatctaccta

B-jararaca_I.3.1 ------------------------------------------------------------

B-jararaca_D.10 ------------------------------------------------------------

B-jararaca_D.07 ------------------------------------------------------------

B-atrox taacatcttttctaatcaatttatattttcacttctattcataaaattgattgaatcact

C-horridus ------------------------------------------------------------

C-viridis ------------------------------------------------------------

P-mucrosquamatus taacatcttttctaatcaatttatatttcacttctattcataaaaaatgattgaatcatt

B-jararaca_I.3.1 ------------------------------------------------------------

B-jararaca_D.10 ------------------------------------------------------------

B-jararaca_D.07 ------------------------------------------------------------

B-atrox atatatccttttttcaaagttcttattaagcctaattccccctcagtaattcaatcattc

C-horridus ------------------------------------------------------------

C-viridis ------------------------------------------------------------

P-mucrosquamatus atatatccttttttcaatattcttattaagcctaatt-tccctcagtatttcaatcattc

B-jararaca_I.3.1 ------------------------------------------------------------

B-jararaca_D.10 ------------------------------------------------------------

B-jararaca_D.07 ------------------------------------------------------------

B-atrox ctgaatcctaaatcttttgaaacttaatttgtctttaaagttcaacttttctttcacctt

C-horridus ------------------------------------------------------------

C-viridis ------------------------------------------------------------

P-mucrosquamatus cttaatcctaaatcttttcaaacttaatttgtctttaaagttcaaacttttctttcactt

B-jararaca_I.3.1 ------------------------------------------------------------

B-jararaca_D.10 ------------------------------------------------------------

B-jararaca_D.07 ------------------------------------------------------------

B-atrox tcatcatcccaatattttta-attttgtatcttatcaaaaa-tactatattacttatatg

C-horridus ------------------------------------------------------------

C-viridis ------------------------------------------------------------

P-mucrosquamatus tcatcatcccaatatttttacattttgtatcttatcaaaaattactatattacttatatc

B-jararaca_I.3.1 ------------------------------------------------------------

B-jararaca_D.10 ------------------------------------------------------------

B-jararaca_D.07 ------------------------------------------------------------

B-atrox tcttatcccacttattctaaagcccctttatagcctaagttatttatttatttagccata

C-horridus ------------------------------------------------------------

C-viridis ------------------------------------------------------------

P-mucrosquamatus tcttatcccacttattctaaagcccctttatagccaaagttatttatttatttagccata

B-jararaca_I.3.1 ------------------------------------------------------------

B-jararaca_D.10 ------------------------------------------------------------

B-jararaca_D.07 ------------------------------------------------------------

B-atrox ttcaaacaactgtttgctcatataatattggat---------------------------

C-horridus ---------atgtttgctcgtataatagtccatctggtagggtgagtcaagtctcttgag

C-viridis ------------------------------------------------------------

P-mucrosquamatus ttcaaacaagtgtttgctcgtataatattccatctggtagggcgagtcaagtctcttgag

B-jararaca_I.3.1 ------------------------------------------------------------

B-jararaca_D.10 ------------------------------------------------------------

B-jararaca_D.07 ------------------------------------------------------------

B-atrox ------------------------------------------------------------

C-horridus cccatctctaatctttttcctttgccaaattgttttccagattaatatttttgacttgtc

C-viridis ------------------------------------------------------------

P-mucrosquamatus cccatctctaatcttcttcctttgccaaattgctttccagattaatattttcgacttgtc

B-jararaca_I.3.1 ------------------------------------------------------------

B-jararaca_D.10 ------------------------------------------------------------

B-jararaca_D.07 ------------------------------------------------------------

B-atrox ------------------------------------------------------------

C-horridus aaacaattcttgtatttttgtagttgttgtttggtgtaaaagatacataatacgcaagct

C-viridis ------------------------------------------------------------

P-mucrosquamatus aaatatttcttgtatttttgtagttgttgttaggcgtagaggatacataatacacaagat

B-jararaca_I.3.1 ------------------------------------------------------------

B-jararaca_D.10 ------------------------------------------------------------

B-jararaca_D.07 ------------------------------------------------------------

B-atrox ------------------------------------------------------------

C-horridus gtatactttttgtctcccaaaaaatgattcttctaacagtatccattttgttaagtcttc

C-viridis ------------------------------------------------------------

P-mucrosquamatus gtatactttttgtctcccaaactgtgattctcctaacagtgtccattttgttaagtcttc

B-jararaca_I.3.1 ------------------------------------------------------------

B-jararaca_D.10 ------------------------------------------------------------

B-jararaca_D.07 ------------------------------------------------------------

B-atrox ------------------------------------------------------------

C-horridus ttttttctcaggtttattttgatgttctaatgtataaggtatcttttccatat-------

C-viridis ------------------------------------------------------------

P-mucrosquamatus tttcttctcaggtttattttgacgttctaatgcataaggtatcttttccatatttattta

B-jararaca_I.3.1 ------------------------------------------------------------

B-jararaca_D.10 ------------------------------------------------------------

B-jararaca_D.07 ------------------------------------------------------------

B-atrox ---cgtatttccattat-------------------------------------------

C-horridus ttccatatttccatattgtttttttt---------------------------atttttg

C-viridis ------------------------------------------------------------

P-mucrosquamatus ttccatatttccatattgtgtgttttttcttatttgtattaagttataattccatttgtg

B-jararaca_I.3.1 ------------------------------------------------------------

B-jararaca_D.10 ------------------------------------------------------------

B-jararaca_D.07 ------------------------------------------------------------

B-atrox -----------------------aattttttcttatttgtattaagttat----------

C-horridus actttgccaat------taagtcatttttttctagtccacattctgttatctgatttgtt

C-viridis ------------------------------------------------------------

P-mucrosquamatus actttgccaattaccggtaagtcatttttttctagttcacattctgttatttgatttgtt

B-jararaca_I.3.1 ------------------------------------------------------------

B-jararaca_D.10 ------------------------------------------------------------

B-jararaca_D.07 ------------------------------------------------------------

B-atrox --------------------------aatttgtgt--gctggaactgtatgttagaat--

C-horridus tgttcattctttacca--attctttgaattttttt--attgtaaatttaagttagaagtg

C-viridis ------------------------------------------------------------

P-mucrosquamatus tgttcattctttaccatcattctttgaatgtgttttgattgtaaatttaagttagaagtg

B-jararaca_I.3.1 ------------------------------------------------------------

B-jararaca_D.10 ------------------------------------------------------------

B-jararaca_D.07 ------------------------------------------------------------

B-atrox --gtttttggcatttaaagggttaagcactgtctccttgagagatggc----ttatgggt

C-horridus cattcatttatgttgtcaaagttatgtttaatctcctcgtgctttagcgctattgtgatt

C-viridis ------------------------------------------------------------

P-mucrosquamatus tattcatttatgttgtcaaagttatgtttaatctcctcatggtttagagctattgtgatt

B-jararaca_I.3.1 ------------------------------------------------------------

B-jararaca_D.10 ------------------------------------------------------------

B-jararaca_D.07 ------------------------------------------------------------

B-atrox aattgtagttttagtgttcagtgaatcctatgttaaaaagtcagtgg-------------

C-horridus tgtatctgttgtagcgttgcataaatttctttttggaaagtatttgtcttgtcttgaaat

C-viridis ------------------------------------------------------------

P-mucrosquamatus tgtatctgtcgtagcattgcatacatttcttcttggaaagtatttgtcttgtcttgaaat

B-jararaca_I.3.1 ------------------------------------------------------------

B-jararaca_D.10 ------------------------------------------------------------

B-jararaca_D.07 ------------------------------------------------------------

B-atrox ------------------------------------------------------------

C-horridus gttgcttctagaggtctttga----tctagctaaatccttcaagagtagatattaagtgt

C-viridis ------------------------------------------------------------

P-mucrosquamatus tttgcttctagaggtctttgatctttctggctaaatctttcaagggacaagaagtctcaa

B-jararaca_I.3.1 ------------------------------------------------------------

B-jararaca_D.10 ------------------------------------------------------------

B-jararaca_D.07 ------------------------------------------------------------

B-atrox ------------------------------------------------------------

C-horridus ggtttt------------------------------------------------------

C-viridis ------------------------------------------------------------

P-mucrosquamatus aatttttgttgcttaagtgaaaaatctgtttagtgagttttgtcccattttacgaccttt

B-jararaca_I.3.1 ------------------------------------------------------------

B-jararaca_D.10 ------------------------------------------------------------

B-jararaca_D.07 ------------------------------------------------------------

B-atrox ------------------------------------------------------------

C-horridus ------------------------------------------------------------

C-viridis ------------------------------------------------------------

P-mucrosquamatus cttgctacagtggttaagtcactcactactattgttaagttagtaacactgttgttaaat

B-jararaca_I.3.1 ------------------------------------------------------------

B-jararaca_D.10 ------------------------------------------------------------

B-jararaca_D.07 ------------------------------------------------------------

B-atrox ------------------------------------------------------------

C-horridus ------------------------------------------------------------

C-viridis ------------------------------------------------------------

P-mucrosquamatus gaatctggcttcttcattgacttcacttgtcagaagtgatcacacaacccagggacactg

B-jararaca_I.3.1 ------------------------------------------------------------

B-jararaca_D.10 ------------------------------------------------------------

B-jararaca_D.07 ------------------------------------------------------------

B-atrox ------------------------------------------------------------

C-horridus ------------------------------------------------------------

C-viridis ------------------------------------------------------------

P-mucrosquamatus caactgtcataaatatgagtcacatgctaagtatctgaacttttttcacatcactgtggg

B-jararaca_I.3.1 ------------------------------------------------------------

B-jararaca_D.10 ------------------------------------------------------------

B-jararaca_D.07 ------------------------------------------------------------

B-atrox ------------------------------------------------------------

C-horridus ------------------------------------------------------------

C-viridis ------------------------------------------------------------

P-mucrosquamatus aatgctgcaatggtcatgtgaaaaactgaatagcatgagcctttctgatggctgagatct

B-jararaca_I.3.1 ------------------------------------------------------------

B-jararaca_D.10 ------------------------------------------------------------

B-jararaca_D.07 ------------------------------------------------------------

B-atrox -----------------gaggggctaccaagttcatcagatgatatcttggctacatgcc

C-horridus -----ctgtcattttttgatgaa-tatgtcata-tccatgttatctccccctttcgagta

C-viridis ------------------------------------------------------------

P-mucrosquamatus aaggcatctggtcttttgagaagctgtggcatattttgcttcacttctccgctgctcagt

B-jararaca_I.3.1 -----atctggtcttatgagaagctgtggcatattttgtttcacttctccactgctcagt

B-jararaca_D.10 -----atctggtcttatgagaagctgtggcatattttgtttcacttctccactgctcagt

B-jararaca_D.07 -----atctggtcttatgagaagctgtggcatattttgtttcacttctccactgctcagt

B-atrox agcttgtaaaagaagcatttg---------------------------------------

C-horridus atccgtcgtaagtgccatagtctctgtaattagtcttgtcaagctggccaaaactcaaaa

C-viridis --ctttctcaagtg----------------------------------------------

P-mucrosquamatus aactttcttcagtgccattgt------------------------------------aaa

B-jararaca_I.3.1 aactttcttcagtgccattgt------------------------------------aaa

B-jararaca_D.10 aactttcttcagtgccattgt------------------------------------aaa

B-jararaca_D.07 aactttcttcagtgccattgt------------------------------------aaa

* **

B-atrox tgaagaaagaca---------------------cgaggtg--------atatgtc-----

C-horridus tccaaacagtccaattttgtatagttcttatctttatatataatttttataatccactgt

C-viridis ------------------------------------------------------------

P-mucrosquamatus tttgaatggtca---------------------ctaaatg-aattgttgtaagtc-----

B-jararaca_I.3.1 tttaaatggtca---------------------ctaaatg-aattgttgtaagtc-----

B-jararaca_D.10 tttaaatggtca---------------------ctaaatg-aattgttgtaagtc-----

B-jararaca_D.07 tttaaatggtca---------------------ctaaatg-aattgttgtaagtc-----

B-atrox ---------------------ctataagagccatggg------------gtcacttc---

C-horridus aattttcaattgtcaaggtttcagtaaaatcaatgttttataacaattctttctttcagg

C-viridis -------------------------------tatgtgtgtgcatggcttttttttt----

P-mucrosquamatus -------------------------aagaactacctgtattaataactagttcctt----

B-jararaca_I.3.1 -------------------------aagaactacctgtattaataactagttcctc----

B-jararaca_D.10 -------------------------aagaactacctgtattaataactagttcctc----

B-jararaca_D.07 -------------------------aagaactacctgtattaataactagttcctc----

* * *

B-atrox -----agaatggagaaggtatgagttg---------------------------------

C-horridus agtcaattgt-caagcagtcagaattgcaattcaatatatttagacaccttgaatccttc

C-viridis ------------------------------------------------------------

P-mucrosquamatus -----actgtgcagggagactgagtggaaagt---------------actaaacatctgt

B-jararaca_I.3.1 -----actgtgcagggaggctgagtagaaagt---------------actaaacatctgt

B-jararaca_D.10 -----actgtgcagggaggctgagtggaaagt---------------actaaacatctgt

B-jararaca_D.07 -----actgtgcagggaggctgagtggaaagt---------------actaaacatctgt

B-atrox --------ttcaccagaagaactc------------------------------------

C-horridus ttgtaactttccctctcaaaagttagtcatagatcagagcttttaaacaattcataaatt

C-viridis ------------------------------------------------------------

P-mucrosquamatus ctccagctttgcttgtacaagctt------------------------------------

B-jararaca_I.3.1 ttccagctttgcttgtacaagctt------------------------------------

B-jararaca_D.10 ttccagctttgcttgtacaagctt------------------------------------

B-jararaca_D.07 ttccagctttgcttgtacaagctt------------------------------------

B-atrox ---aacaagactaagttattgtttgaggttc--------ctgatgatttaaatgatccaa

C-horridus tagaccataatcaaagtatttcttttccttcaaaacactttaaagtcttatag-----ct

C-viridis ----------------------ttttgtcatagaac------------------------

P-mucrosquamatus ----------tcacataatggcctttgtcttgcagc---ttgaagatgtatggagttcaa

B-jararaca_I.3.1 ----------tcggattatggcctttgtcttgcagc---ttgaagatgtatggagttcaa

B-jararaca_D.10 ----------tcggattatggcctttgtcttgcagc---ttgaagttgtatggagttcaa

B-jararaca_D.07 ----------tcggattatggcctttgtcttgcagc---ttgaagttgtatggagttcaa

*

B-atrox tattatccca-------------------ttctttggcatttatctgatt---gagaaga

C-horridus t----gcccagtatacaatctctcctttgttccctggcaactatcttcttaaaggttatt

C-viridis -----gcccagaac---------------------------------------gg-----

P-mucrosquamatus t----tcccagaat------tcgccatgctggccggggacttgt---------gggaatt

B-jararaca_I.3.1 t----tcccagaat------tcgccatgctggccggggacttat---------gggaatt

B-jararaca_D.10 t----tcccagaat------tcgccatgctggccagggacttgt---------gg-----

B-jararaca_D.07 t----tcccagaat------tcgccatgctggccagggacttgt---------gg-----

**** *

B-atrox aaagattaaaga------------------------------------------------

C-horridus agagttcatgggaatagatttttctttgtttctgggcattgtctttaatgtgctatattc

C-viridis ------------------------------------------------------------

P-mucrosquamatus gaaatccata-------------------------------------------catcttc

B-jararaca_I.3.1 gaaatccata-------------------------------------------catcttc

B-jararaca_D.10 gaaatccatg-------------------------------------------catcttc

B-jararaca_D.07 gaaatccatg-------------------------------------------catcttc

B-atrox -------------gaaattttgaattaattattt------ttttctagttcacattcact

C-horridus taattgtccactagatgtcagtaaatatttattttccatctctttcaga-------caag

C-viridis ------------------------------------------------------------

P-mucrosquamatus aagttgcc-----aaggttgagaaacactggttt---aattgattgaaagtacaatcagc

B-jararaca_I.3.1 aagttgcc-----aaggttaagaaacactggttt---aattggttgaaagtacaatcagt

B-jararaca_D.10 aagttgtc-----aaggttgagaaacactggttt---aattggttgaaagtacaatcagc

B-jararaca_D.07 aagttgtc-----aaggttgagaaacactggttt---aattggttgaaagtacaatcagc

B-atrox tatctgatttgtttgttcatt-gtttac--------------------------------

C-horridus ttcttattatttttctttatatatatatatatatatatatatatatatatatatatatat

C-viridis ------------------------------------------------------------

P-mucrosquamatus tcatttatattgtggtttata-agatgctcttaggacagtgatgacgaaccttttagaga

B-jararaca_I.3.1 tcattgatattgtggttcata-agatgc--------------------------------

B-jararaca_D.10 tcattgatattgtggttcata-agatgc--------------------------------

B-jararaca_D.07 tcattgatattgtggttcata-agatgc--------------------------------

B-atrox ------------------------------------------------------------

C-horridus atatatatatatatatatatatatatatatatatatatannnnnnnnnnnnnnnnnnnnn

C-viridis ------------------------------------------------------------

P-mucrosquamatus ccgagtgcccaaactgcaacccaaagcccatgatttat----------------------

B-jararaca_I.3.1 ------------------------------------------------------------

B-jararaca_D.10 ------------------------------------------------------------

B-jararaca_D.07 ------------------------------------------------------------

B-atrox ------------------------------------------------------------

C-horridus nnnnnnnnnnnnnnnnnnnnnnnnnnnnnnnnnnnnnnnnnnnnnnnnnnnnnnnnnnnn

C-viridis ------------------------------------------------------------

P-mucrosquamatus ------------------------------------------------------------

B-jararaca_I.3.1 ------------------------------------------------------------

B-jararaca_D.10 ------------------------------------------------------------

B-jararaca_D.07 ------------------------------------------------------------

B-atrox ------------------------------------------------------------

C-horridus nnnnnnnnnnnnnnnnnnnnnnnnnnnnnnnnnnnnnnnnnnnnnnnnnnnnnnnnnnnn

C-viridis ------------------------------------------------------------

P-mucrosquamatus ------------------------------------------------------------

B-jararaca_I.3.1 ------------------------------------------------------------

B-jararaca_D.10 ------------------------------------------------------------

B-jararaca_D.07 ------------------------------------------------------------

B-atrox ------------------------------------------------------------

C-horridus nnnnnnnnnnnnnnnnnnnnnnnnnnnnnnnnnnnnnnnnnnnnnnnnnnnnnnnnnnnn

C-viridis ------------------------------------------------------------

P-mucrosquamatus ------------------------------------------------------------

B-jararaca_I.3.1 ------------------------------------------------------------

B-jararaca_D.10 ------------------------------------------------------------

B-jararaca_D.07 ------------------------------------------------------------

B-atrox ------------------------------------------------------------

C-horridus nnnnnnnnnnnnnnnnnnnnnnnnnnnnnnnnnnnnnnnnnnnnnnnnnnnnnnnnnnnn

C-viridis ------------------------------------------------------------

P-mucrosquamatus ------------------------------------------------------------

B-jararaca_I.3.1 ------------------------------------------------------------

B-jararaca_D.10 ------------------------------------------------------------

B-jararaca_D.07 ------------------------------------------------------------

B-atrox ------------------------------------------------------------

C-horridus nnnnnnnnnnnnnnnnnnnnnnnnnnnnnnnnnnnnnnnnnnnnnnnnnnnnnnnnnnnn

C-viridis ------------------------------------------------------------

P-mucrosquamatus ------------------------------------------------------------

B-jararaca_I.3.1 ------------------------------------------------------------

B-jararaca_D.10 ------------------------------------------------------------

B-jararaca_D.07 ------------------------------------------------------------

B-atrox ------------------------------------------------------------

C-horridus nnnnnnnnnnnnnnnnnnnnnnnnnnnnnnnnnnnnnnnnnnnnnnnnnnnnnnnnnnnn

C-viridis ------------------------------------------------------------

P-mucrosquamatus ------------------------------------------------------------

B-jararaca_I.3.1 ------------------------------------------------------------

B-jararaca_D.10 ------------------------------------------------------------

B-jararaca_D.07 ------------------------------------------------------------

B-atrox ------------------------------------------------------------

C-horridus nnnnnnnnnnnnnnnnnnnnnnnnnnnnnnnnnnnnnnnnnnnnnnnnnnnnnnnnnnnn

C-viridis ------------------------------------------------------------

P-mucrosquamatus ---------------------caccgggtgcaaggtgggtgtggcccactggatgggcgt

B-jararaca_I.3.1 ------------------------------------------------------------

B-jararaca_D.10 ------------------------------------------------------------

B-jararaca_D.07 ------------------------------------------------------------

B-atrox ------------------------------------------------------------

C-horridus nnnnnnnnnnnnnnnnnnnnnnnnnnnnnnnnnnnnnnnnnnnnnnnnnnnnnnnnnnnn

C-viridis ------------------------------------------------------------

P-mucrosquamatus ggctgtggtggtcatggcacactgggtgtggccaaggtgggcgtgatcatctcaatgttg

B-jararaca_I.3.1 ------------------------------------------------------------

B-jararaca_D.10 ------------------------------------------------------------

B-jararaca_D.07 ------------------------------------------------------------

B-atrox ------------------------------------------------------------

C-horridus nnnnnnnnnnnnnnnnnnnnnnnnnnnnnnnnnnnnnnnnnnnnnnnnnnnnnnnnnnnn

C-viridis ------------------------------------------------------------

P-mucrosquamatus ctcgtggggagggatgcctgtgggggtgggcagggttgggatagatgggacgataggcag

B-jararaca_I.3.1 ------------------------------------------------------------

B-jararaca_D.10 ------------------------------------------------------------

B-jararaca_D.07 ------------------------------------------------------------

B-atrox ------------------------------------------------------------

C-horridus nnnnnnnnnnnnnnnnnnnnnnnnnnnnnnnnnnnnnnnnnnnnnnnnnnnnnnnnnnnn

C-viridis ------------------------------------------------------------

P-mucrosquamatus gaagctcgttctgaggggtgtggtgaggcagggtgggggtgctattgacatggggcaagg

B-jararaca_I.3.1 ------------------------------------------------------------

B-jararaca_D.10 ------------------------------------------------------------

B-jararaca_D.07 ------------------------------------------------------------

B-atrox ------------------------------------------------------------

C-horridus nnnnnnnnnnnnnnnnnnnnnnnnnnnnnnnnnnnnnnnnnnnnnnnnnnnnnnnnnnnn

C-viridis ------------------------------------------------------------

P-mucrosquamatus caagggctggggcagctgactgacaccattggcccctgtacaaagaggccagtgaacaca

B-jararaca_I.3.1 ------------------------------------------------------------

B-jararaca_D.10 ------------------------------------------------------------

B-jararaca_D.07 ------------------------------------------------------------

B-atrox ------------------------------------------------------------

C-horridus nnnnnnnnnnnnnnnnnnnnnnnnnnnnnnnnnnnnnnnnnnnnnnnnnnnnnnnnnnnn

C-viridis ------------------------------------------------------------

P-mucrosquamatus tggggaccgctgtcatccgttagcagccacagccagctggcccctcaactggtgcactct

B-jararaca_I.3.1 ------------------------------------------------------------

B-jararaca_D.10 ------------------------------------------------------------

B-jararaca_D.07 ------------------------------------------------------------

B-atrox ------------------------------------------------------------

C-horridus nnnnnnnnnnnnnnnnnnnnnnnnnnnnnnnnnnnnnnnnnnnnnnnnnnnnnnnnnnnn

C-viridis ------------------------------------------------------------

P-mucrosquamatus ggggctctgcagcccgccgtcagcagttcaaagaagccgcctctgagctggatagccggc

B-jararaca_I.3.1 ------------------------------------------------------------

B-jararaca_D.10 ------------------------------------------------------------

B-jararaca_D.07 ------------------------------------------------------------

B-atrox ------------------------------------------------------------

C-horridus nnnnnnnnnnnnnnnnnnnnnnnnnnnnnnnnnnnnnnnnnnnnnnnnnnnnnnnnnnnn

C-viridis ------------------------------------------------------------

P-mucrosquamatus cgacagctaaggaggtgatgcagtgggtcatgcccaggaaaaggagagcatgtgaggcca

B-jararaca_I.3.1 ------------------------------------------------------------

B-jararaca_D.10 ------------------------------------------------------------

B-jararaca_D.07 ------------------------------------------------------------

B-atrox ------------------------------------------------------------

C-horridus nnnnnnnnnnnnnnnnnnnnnnnnnnnnnnnnnnnnnnnnnnnnnnnnnnnnnnnnnnnn

C-viridis ------------------------------------------------------------

P-mucrosquamatus ggagggatgggacgggggggagggaaaccagctgtgccgggatgcatccagccacatggg

B-jararaca_I.3.1 ------------------------------------------------------------

B-jararaca_D.10 ------------------------------------------------------------

B-jararaca_D.07 ------------------------------------------------------------

B-atrox ------------------------------------------------------------

C-horridus nnnnnnnnnnnnnnnnnnnnnnnnnnnnnnnnnnnnntaatatatatatatatatatata

C-viridis ------------------------------------------------------------

P-mucrosquamatus actttggcgagttagcaggcggctggttgaagagggacagttgccatctccggggcatgg

B-jararaca_I.3.1 ------------------------------------------------------------

B-jararaca_D.10 ------------------------------------------------------------

B-jararaca_D.07 ------------------------------------------------------------

B-atrox ------------------------------------------------------------

C-horridus tatatatatatatatatatatatatatatatatatatatatatatatatatatatatata

C-viridis ------------------------------------------------------------

P-mucrosquamatus tacatgtgcccacagagaggactctgagtgc-----------------------cgcctc

B-jararaca_I.3.1 ------------------------------------------------------------

B-jararaca_D.10 ------------------------------------------------------------

B-jararaca_D.07 ------------------------------------------------------------

B-atrox ------------------------------------------------------------

C-horridus tataatttaatcaaaatcctttagaatataatcttagcaatttgttttagtttctatgtt

C-viridis ------------------------------------------------------------

P-mucrosquamatus tggcacccgtgccataggtttgccaccactgtcttaggagcttctat-------------

B-jararaca_I.3.1 -------------------------------tctaaggaacttctat-------------

B-jararaca_D.10 -------------------------------tctaaggaacttctat-------------

B-jararaca_D.07 -------------------------------tctaaggaacttctat-------------

B-atrox --------------------catcattctttgaatgtgt------tttgattgtaaattt

C-horridus tgatcagattgtagtattttaaatttaatttcaatatgtttctttcctccttaaaggtta

C-viridis ------agttctggcag-------------------------------------------

P-mucrosquamatus ------agttccagcagcaagaccctgctttaaataactacgatcctggagtgagaattc

B-jararaca_I.3.1 ------agttccagcagcaataccctgctttaaataactaagatcctgcagtgagaattc

B-jararaca_D.10 ------agttccagcagcaataccctgctttaaataactaagatcctgcagtgagaattc

B-jararaca_D.07 ------agttccagcagcaataccctgctttaaataactaagatcctgcagtgagaattc

B-atrox a--------------------------agtaagaaagtgtattcatttgtgttgtcaaag

C-horridus atttgccacttctgtctgtggggaaatgagaatatagtgccctccccttactttcaga--

C-viridis ------------------------------------------------------------

P-mucrosquamatus a--------------------gggggtgataaaagtgtctgtttcacttccctattga--

B-jararaca_I.3.1 a--------------------gggggtgataaaagggtctgtttcccttccttttgaa--

B-jararaca_D.10 a--------------------gggggtgataaaagggtctgtttcacttcccttttga--

B-jararaca_D.07 a--------------------gggggtgataaaagggtctgtttcacttcccttttga--

B-atrox ttatgtttaatctcctctggtttagaa---------------------------------

C-horridus --gg--------ttttttgctttggggatctttcccagggacttagttgcaaaataaggg

C-viridis ----------------cttttttgga----------------------------------

P-mucrosquamatus --ataagtcaactctcctgttttggaaatctttcc-------------------------

B-jararaca_I.3.1 ---taagtcaactctcctgttttggaaatctttct-------------------------

B-jararaca_D.10 --ataagtcaactctcctgttttggaaatctttct-------------------------

B-jararaca_D.07 --ataagtcaactctcctgttttggaaatctttct-------------------------

* *** *

B-atrox --------cagggtgtcaaactcaatttca----------ttgagggccgcatcagcagg

C-horridus agaaaagatcaaatactctcaccaatttcaaatacattgttcactgttgttcttgttaga

C-viridis ------------------------------------------------------------

P-mucrosquamatus ----atgcccaaatcttatcaccactaacaaa--------gcaaagcctgcttcactaaa

B-jararaca_I.3.1 ----acgcccaaatcgtatcaccactaacaaa--------gcaaagcctgcttcactaaa

B-jararaca_D.10 ----acgcccaaatcttatcaccactaacaaa--------gcaaagcctgcttcactaaa

B-jararaca_D.07 ----acgcccaaatcttatcaccactaacaaa--------gcaaagcctgcttcactaaa

B-atrox tggttgc------------------------------------cctggggggctgggtgg

C-horridus gctctgggttttcgg------ctacaggtagcttgggctagaggctggcatgtggctagt

C-viridis --------------------------------------tggaaccttgtaggatag----

P-mucrosquamatus gtgttaagtgttaagaagaccctggtcatatcttatttcagagcacggtgagatagctgt

B-jararaca_I.3.1 gtgttaagtgttaagaagaccctggtcatatcttatttcagagcttggtgagctagctgt

B-jararaca_D.10 gtgttaagtgttaagaagaccctggtcatatcttatttcagagcttggtgagctagctgt

B-jararaca_D.07 gtgttaagtgttaagaagaccctggtcatatcttatttcagagcttggtgagctagctgt

* *

B-atrox gtgtggcttactgggtgggtgggaatt---cgaattccat--------------------

C-horridus ctttcttctgacgggt---------ttggagggctttccctgtatcaaacgaagcaattg

C-viridis ------------------------------------------------------------

P-mucrosquamatus gtaatatttaatgaat---------tt---ttgctttcctaaaacca-------------

B-jararaca_I.3.1 gtaaaatttaatgaat---------tt---tggctttcat-aaatca-------------

B-jararaca_D.10 gtaaaatttaatgaat---------tt---tggctttcat-aaatca-------------

B-jararaca_D.07 gtaaaatttaatgaat---------tt---tggctttcat-aaatca-------------

B-atrox ------------------------------------------------------------

C-horridus ccgatgctttgtgatcaacgatgcacccttccttcacaatcacctcttcggggcgacttc

C-viridis ------------------------------------------------------------

P-mucrosquamatus ------------------------------------------------------------

B-jararaca_I.3.1 ------------------------------------------------------------

B-jararaca_D.10 ------------------------------------------------------------

B-jararaca_D.07 ------------------------------------------------------------

B-atrox ----------------------------------ttgtaagggaaatgggaattaaattt

C-horridus agacccatctagggtccccttggaaggacgaaattagctgtggggagcggagatcca-tt

C-viridis ------------------------------------------gcaaggcgggttgta--t

P-mucrosquamatus ----------------------------------tgtttgaagaaatgggaattaaa-tt

B-jararaca_I.3.1 ----------------------------------tatttgaaggaatgggaattaaa-tt

B-jararaca_D.10 ----------------------------------tatttgaaggaatgggaattaaa-tt

B-jararaca_D.07 ----------------------------------tatttgaaggaatgggaattaaa-tt

* * * * * *

B-atrox ttcctatg--------------------------------------ttgttatgttaaac

C-horridus ctcacatagccatgcgctacgggtgcaaaactagaagtctcaaaatttttgttgcttaac

C-viridis gacatgtg----------------------------------------------------

P-mucrosquamatus ttcttatg--------------------------------------ttgttatattaaac

B-jararaca_I.3.1 ttcctatg--------------------------------------ttgttatgttaaac

B-jararaca_D.10 ttcctatg--------------------------------------ttgttatgttaaac

B-jararaca_D.07 ttcctatg--------------------------------------ttgttatgttaaac

* *

B-atrox aataagatc---------------------------------------------------

C-horridus tgaaaaatctgtttagtgagttttgtcccattttatgacctttcttgctacagtggttaa

C-viridis ------------------------------------------------------------

P-mucrosquamatus aagaagat----------------------------------------------------

B-jararaca_I.3.1 aagaagat----------------------------------------------------

B-jararaca_D.10 aagaagat----------------------------------------------------

B-jararaca_D.07 aagaagat----------------------------------------------------

B-atrox ----ctcaaactgagtttttatcttaagataag-----ataacctttattatctcttttc

C-horridus gtcactcaaactgagttcttatcttaagataagacaggataaactttattgtcacttttc

C-viridis -----------tgagttcc------------------ggcatcttttatt-----tttac

P-mucrosquamatus ----ctctaactgacttcttatcttaagataagatagaataacctttattgtca-ttttc

B-jararaca_I.3.1 ----ctcagactgagttcttatcttaagataag-----ataacctttattatcacttttc

B-jararaca_D.10 ----ctcagactgagttcttatcttaagataag-----ataacctttattatcacttttc

B-jararaca_D.07 ----ctcagactgagttcttatcttaagataag-----ataacctttattatcacttttc

*** ** * ****** *** *

B-atrox tgtgaacacactggcacaggttaaaaatgaatggctgatgccctttctgaaaattgtat-

C-horridus tatgaacatactggcacacgttaaaaatgaaattctgatgccctttctcaaaagtatatg

C-viridis aaagaa-------gcacaca----------------------------------------

P-mucrosquamatus tatgaacacactggcacatgttaaaaatgaaattctgatgctctttctcaaaagtatatg

B-jararaca_I.3.1 tgtgaacacactggcacacgttaaaaatgaaattccgatgccctttctgaaaagtgtatg

B-jararaca_D.10 tgtgaacacactggcacacgttaaaaatgaaattccgatgccctttctgaaaagtgtatg

B-jararaca_D.07 tgtgaacacactggcacacgttaaaaatgaaattccgatgccctttctgaaaagtgtatg

*** *****

B-atrox ----tgtatatacacacac-------------aattaatcactaga---tcaca-gtagt

C-horridus tgtgtgtatatacatataa--acacacacaaatattaatcacttaagattcacaagtaat

C-viridis ----cacacacacacacacacacacactcacatattaatcacttaagatttacaggccgt

P-mucrosquamatus tgtgtacacacacacacacacacacacacacaaattaatcacttaagattcacaggtagt

B-jararaca_I.3.1 tgtgtgtatatacacacac-------------aattaatcacttaagattcacaggtagt

B-jararaca_D.10 tgtgtgtatatacacacac-------------aattaatcacttaagattcacaggtagt

B-jararaca_D.07 tgtgtgtatatacacacac-------------aattaatcacttaagattcacaggtagt

* * *** * * ********** * * *** * *

B-atrox tt-----------------------acctacatgagcataggcatatatatgatttgcaa

C-horridus tt-----------------------acctacatgggcataggcacatatatgatttccaa

C-viridis ttaaataaataaaataaataaataaatctacatgggcataggcatatatatgatttgcaa

P-mucrosquamatus tt-----------------------acctacatgggcataggcatatatatgatttgcaa

B-jararaca_I.3.1 tt-----------------------acctacatgggcataggcatatatctgatttgcaa

B-jararaca_D.10 tt-----------------------acctacatgggcataggcatatatctgatttgcaa

B-jararaca_D.07 tt-----------------------acctacatgggcataggcatatatctgatttgcaa

** * ******* ********* **** ****** ***

B-atrox ataatccattaataacaactggtttaatgct-tgttgtccacagtcatctg-ttacccct

C-horridus ataatccattttattcaactggtttgatgctgtgtgtcccacagtcatctgtttacccat

C-viridis ataatccattttattctactagtttgatgctgtgtgtcccacaaccatctg-ttaccctc

P-mucrosquamatus ataatccattttattcaactggtttgatgatgtgtgtcccacagtcatctg-ttaccctc

B-jararaca_I.3.1 ataatccattttattcaactggtttgatgctgtgtgtcccacagtcatctg-ttacccct

B-jararaca_D.10 ataatccattttattcaactggtttgatgctgtgtgtcccacagtcatctg-ttacccct

B-jararaca_D.07 ataatccattttattcaactggtttgatgctgtgtgtcccacagtcatctg-ttacccct

********** * *** **** *** * *** ***** ****** ******

B-atrox caaaattagtgtactgatagaaataatatcaataaaagcaattttgttctactccacagc

C-horridus caaaattagtgtactgatagaaacactaccaataaaagtgattctgttctactccacagc

C-viridis caaaattagtttactgatagaaacactaccaataaaagtgattctgttctactccacagc

P-mucrosquamatus cataattagtgtattgatggaaacactaccaataaaagcgattctgttctactccacagt

B-jararaca_I.3.1 caaaattagtgtactgatagaaacactaccaataaaagcaattttgttctactccacagc

B-jararaca_D.10 caaaattagtgtactgatagaaacactaccaataaaagcaattttgttctactccacagc

B-jararaca_D.07 caaaattagtgtactgatagaaacactaccaataaaagcaattttgttctactccacagc

** ******* ** **** **** * ** ********* *** ***************

Exon 2

B-atrox caaaca-tgaacctttttgatttctttacagCACAAAAGTCTTCTGAACTGGTCATTGGA

C-horridus caaacactgaacgtttttggtttctttgtagCACAAAAGTCTTCTGAACTGGTCGTTGGA

C-viridis caaacactgaacgtttttggttt-tttgcagCACAAAAGTCTTCTGAACTGGTCATTGGA

P-mucrosquamatus caaacactgaacttttttggtttctttgcagCACAAAAGTCTTCTGAACTGGTCATTGGA

B-jararaca_I.3.1 caaacactgaacctttttgatttctttgcagCACAAAAGTCTTCTGAACTGGTCGTTGGA

B-jararaca_D.10 caaacactgaacctttttgatttctttgcagCACAAAAGTCTTCTGAACTGGTCGTTGGA

B-jararaca_D.07 caaacactgaacctttttgatttctttgcagCACAAAAGTCTTCTGAACTGGTCGTTGGA

****** ***** ****** *** *** ************************* *****

Cys

B-atrox GGTGATGAA**TGT**GACATAAATGAACATCCTTTCCTTGCATTCATGTACTACTCTCCCCGG

C-horridus GGTGATGAA**TGT**AACATAAATGAACATCGTTCCCTTGTACTCGTGT---ACTCTGACGGG

C-viridis GGTGATGAA**TGT**AACATAAATGAACATCGTTTCCTTGTAGCCTTGTATGACCCTGACGGG

P-mucrosquamatus GGTGATGAA**TGT**AACATAAATGAACATCCTTTCCTTGTACTCGTGT---ACTATGATGAT

B-jararaca_I.3.1 GGTGATGAA**TGT**GACATAAATGAACATCCTTTCCTTGCATTCCTGT---ACTCTCACGGG

B-jararaca_D.10 GGTGATGAA**TGT**GACATAAATGAACATCCTTTCCTTGCATTCCTGT---ACTCTCACGGG

B-jararaca_D.07 GGTGATGAA**TGT**GACATAAATGAACATCCTTTCCTTGCATTCCTGT---ACTCTCACGGG

************ *************** ** ***** * * *** ** *

Cys HisCys

B-atrox TATTTC**TGT**GGTATGACTTTGATCAACCAGGAATGGGTGCTGACCGCTGCA**CACTGT**AAC

C-horridus ATTCAA**TGT**GGTGGGACTTTGATCAACCAGGAATGGATGCTCACGGCTGCA**CACTGC**GAC

C-viridis TTTCTC**TGT**GGTGGGATTTTGCTCAACGAGGAATGGGTGCTCACTGCTGCA**CACTGC**GAC

P-mucrosquamatus TATCAA**TGC**GGTGGGACTTTGCTCAATGAGGAATGGGTGCTCACTGCTGCA**CACTGC**AAT

B-jararaca_I.3.1 TATTTC**TGT**GGTTTGACTTTGATCAACCAGGAATGGGTGCTGACCGCTGCA**CACTGT**GAC

B-jararaca_D.10 TATTTC**TGT**GGTTTGACTTTGATCAACCAGGAATGGGTGCTGACCGCTGCA**CACTGT**GAC

B-jararaca_D.07 TATTTC**TGT**GGTTTGACTTTGATCAACCAGGAATGGGTGCTGACCGCTGCA**CACTGT**GAC

* ** *** ** **** **** ******** **** ** *********** *

Intron 2

B-atrox AGgtgagggcattacagaatgtggcag-gttga-ggaaatcgtgtttagatatctacatt

C-horridus GTgtgagggcattatagaacatggaagtgttgagggaaatcgtgtttagatatccagatt

C-viridis AGgtgagggcataacagaatatggaagggttgagggaaatcgtgtttagatatccagatt

P-mucrosquamatus GGgtgaggacattacagaatgtggaagggttgagggagatcgtgtttagatatccagatt

B-jararaca_I.3.1 AGgtgagggcattacagaatgtggcagggttgagggaaatcgtgtttagatatctagatt

B-jararaca_D.10 AGgtgagggcattacagaatgtggcagggttgagggaaatcgtgtttagatatctagatt

B-jararaca_D.07 AGgtgagggcattacagaatgtggcagggttgagggaaatcgtgtttagatatctagatt

****** *** * **** *** ** ***** *** **************** * ***

B-atrox tttttataatgtttcagctagtttttagtgacttctctgg--attttgaatgcatgttct

C-horridus tt---------tttctactagtttttagtgacttctctgtgcattttgaatgagtgttct

C-viridis ttttaataatgtttctgctagtttttagtgacttctctgtgcattttgaatgaatgttat

P-mucrosquamatus tttttataatgtttctgctagtttttagtgacttctctgtgcattttgaatgagtgttct

B-jararaca_I.3.1 tatttataatgtttctgctagtttttagtgacttctctgtgcattttgaatgcatgttct

B-jararaca_D.10 tatttataatgtttctgctagtttttagtgactactctgtgcattttgaatgcatgttct

B-jararaca_D.07 tatttataatgtttctgctagtttttagtgactactctgtgcattttgaatgcatgttct

* **** **************** ***** ********** **** *

B-atrox gctgttactgctattgctcttcacaatcatatcttgagcaatgctgtttatatttatggt

C-horridus gctgtaactgttattgctcctcacaatcatatcttgagcaatgctgtttatatttatggt

C-viridis gctgttactgttattgctccacacaataatatcttgaacaatgctgtttatatttatggt

P-mucrosquamatus gctgctactgctattgctcctcacaatcatatcttgagcaatgctgtttaaatatatgat

B-jararaca_I.3.1 gctgttactgctattgctcttcacaatcatatcttgagcaatgctgtttatatttatggt

B-jararaca_D.10 gctgttactgctattgctcttcacaatcatatcttgagcaatgctgtttatatttatggt

B-jararaca_D.07 gctgttactgctattgctcttcacaatcatatcttgagcaatgctgtttatatttatggt

**** **** ******** ****** ********* ************ ** **** *

B-atrox -gaatttccatcatcttcaccatagtattcatacctttccttttttctgggatat-aata

C-horridus tgaatttccatcttcttcaccacagtattcatatctttccttttttctgggataa-aata

C-viridis cgaatttccatcttcttcaccatagtattcatatctttccttttttctgggatat-aata

P-mucrosquamatus tgaatttccatgttcttcaccacagtattcatatctttccttttttctgggatat-aata

B-jararaca_I.3.1 tgaatttccatcatcttcaccatagtattcatatctttccttttttctgggatataaata

B-jararaca_D.10 tgaatttccatcatcttcaccatagtattcatatctttccttttttctgggatat-aata

B-jararaca_D.07 tgaatttccatcatcttcaccatagtattcatatctttccttttttctgggatat-aata

********** ********* ********** ******************** ****

B-atrox ataataataataacaaaatctggaacatttcctgaataccatcagcagtgacaaaatcac

C-horridus ataacaagaataagaaaatctgaaacattacctgaataccatcagcactgacaaaatcac

C-viridis ataacaaaaataacaaaatctggaacattacctgaataccatcaacattgacaaaatcac

P-mucrosquamatus ataacaagaataacaaaatctggaacattacctgaataccaccagcattgacaaaatcac

B-jararaca_I.3.1 ataacaagaataacaaaatctggaacattacctgaataccatcagcactgacaaaatcac

B-jararaca_D.10 ataacaagaataacaaaatgtggaacattacctgaataccatcagcactgacaaaatcac

B-jararaca_D.07 ataacaagaataacaaaatgtggaacattacctgaataccatcagcactgacaaaatcac

**** ** ***** ***** ** ****** *********** ** ** ************

B-atrox cacagttaattgcaaaaggcagccttacttgaa--atcctgtgacaatccctttaatacc

C-horridus cacagtcaattgcaaaaggcagccttacttggaacatcctgtgacaatccctttaatgcc

C-viridis cacagtcaattgccaaaggcatccttacttggaacatcctgtgacaatccctttaatacc

P-mucrosquamatus cacagtcaattgcaaaaggcagccttaattggaacatcttgtggcaatccctttaatacc

B-jararaca_I.3.1 tacagttaattgcaaaaggcagccttacttggaacatcctgtgacaatccctttaatacc

B-jararaca_D.10 tacagttaattgcaaaaggcagccttacttggaacatcctgtgacaatccctttaatacc

B-jararaca_D.07 tacagttaattgcaaaaggcagccttacttggaacatcctgtgacaatccctttaatacc

***** ****** ******* ***** *** * *** **** ************* **

B-atrox atggaaccacaaaatttacctacccca-gtggttgggaaggacttgataggtggataaaa

C-horridus atggaaccacaaaatttacctactccaggtccttgggaagcacttgataggtggataaaa

C-viridis atggaaccacaaaattta-ctaccccaggtccttgggaag-acctgataggtgggtaaaa

P-mucrosquamatus atggaaccacagaatttatctaccccaggtccttgggaaggacttgataggtggataaaa

B-jararaca_I.3.1 atggaaccacaaaatttacctaccccaggtccttgggaaggacttgataggtggataaaa

B-jararaca_D.10 atggaaccacaaaatttacctaccccaggtccttgggaaggacttgataggtggataaaa

B-jararaca_D.07 atggaaccacaaaatttacctaccccaggtccttgggaaggacttgataggtggataaaa

*********** ****** **** *** ** ******** ** ********** *****

B-atrox atgccaaatccagtctaaatatctgactgactgcgcaactaaccagaaaatattaaccat

C-horridus atgccaaatccagtctaaatatctgactgactgtgcaactgaccagaaaatattaactac

C-viridis atgccaaatccagtctaaatatctgactgactatgcaagtaaccagaaaatattaaccat

P-mucrosquamatus atgccaaatccagtctaaatatctgactgactgcgcaactaaccagaaaatagtaaccat

B-jararaca_I.3.1 atgccaaatccagtctaaatatctgactgactgtgcaactaaccagaaaatattaactat

B-jararaca_D.10 atgccaaatccagtctaaatatctgactgactgtgcaactaaccagaaaatattaactat

B-jararaca_D.07 atgccaaatccagtctaaatatctgactgactgtgcaactaaccagaaaatattaactat

******************************** **** * *********** **** *

B-atrox atgctgtctcactctggggg-ctgccataagaaaata-ttttttt--taaaaaatcagta

C-horridus atgctgcctgactgtggggcattgcc--aagaaaatcattttttt--aaaaaaaccagta

C-viridis atgctgcctgactctggaggtttgccataagaaaataaatttaaa--aaaaaaatcagta

P-mucrosquamatus atgctgcctcactctgggggtttgccataa-aaaata-ttttttt--aaaaaaatcagta

B-jararaca_I.3.1 atgctgtttcactctggggg-ctgccataagaaaatatttttttt--taaaaaatcagta

B-jararaca_D.10 atgctgtctcactct-gggggctgccataagaaaata-ttttttt--aaaaaaatcagta

B-jararaca_D.07 atgctgtctcactctggggg-ctgccataagaaaatatttttttttaaaaaaaatcagta

****** * *** * * * **** ** ***** *** ****** *****

B-atrox aaacata-------aaaagttgatgaagtgagaggcttctctctctcccttatcaaaggc

C-horridus caacaaaaaagataaaaagatgatgaagtgagaggct--tctctcttccttatcaaaggc

C-viridis aaacataaaacataaaaagatgatgaagtgagaggct--tctctctcccttaccaaaggc

P-mucrosquamatus aaacataaaagatgaaaagatgatgaagtgagaggct--tctctctcccttatcaaaggc

B-jararaca_I.3.1 aaacata-------aaaaggtgatgaagtgagaggct--tctctctcccttatcaaaggc

B-jararaca_D.10 aaacata-------aaaaggtgatgaagtgagaggct--tctctctcccttatcaaaggc

B-jararaca_D.07 aaacata-------aaaaggtgatgaagtgagaggct--tctctctcccttatcaaaggc

**** * ***** ***************** ******* ***** *******

B-atrox ctgggtgaggcactacatcttaataatacttcttgtaaag-ttgtgcagcgtgggca-ca

C-horridus ctgggtgaggcactacatct---taatacctcttctaaac-aagagcagagtgagcacca

C-viridis ctaggtgaggcactacatct---taatatctcttctaaac-aagagcagagtgggcacca

P-mucrosquamatus ctgggtgaggcactacacct---taatacctcttctaaac-aagagcagagtgggcacca

B-jararaca_I.3.1 ctgggtgaggcactacatct---taatacttcttctaaag-aagagcagagtgggcacca

B-jararaca_D.10 ctgggtgaggcactacatct---taatacttcttctaaag-aagagcagagtgggcacca

B-jararaca_D.07 ctgagtgaggcactacatct---taatacttcttctaaagaaagagcagagtgggcacca

** ************* ** ***** **** **** * **** *** *** **

B-atrox tccagattttttgttctttatgtaccactgttgtgggtatctctggaccagctggggagg

C-horridus tccagattttgtgttcata-----------------------------------------

C-viridis tccagtttttgtgttcgtc--------------------tttatctatcccaccatcctt

P-mucrosquamatus ttcagattttgtgttcgtc-----------------------------------------

B-jararaca_I.3.1 tccagattttgtgttcgtc-----------------------------------------

B-jararaca_D.10 tccagattttgtgttcgtc-----------------------------------------

B-jararaca_D.07 tccagattttgtgttcgtc-----------------------------------------

* *** **** ***** *

B-atrox tgtcggaggatgaggagggggagccggacccctctgagtgtttgccctcccggcagccga

C-horridus ------------------------------------------------------------

C-viridis tcagataggctgggttgagagaggatgtgcgcaatacaaagtcataacattagtaccgtg

P-mucrosquamatus ------------------------------------------------------------

B-jararaca_I.3.1 ------------------------------------------------------------

B-jararaca_D.10 ------------------------------------------------------------

B-jararaca_D.07 ------------------------------------------------------------

B-atrox gccaggcccctctgatgtttgcccctcccgacaccaggcagacagggtggaaatggagtt

C-horridus ------------------------------------------------------------

C-viridis accacatgggttctatctttaacacagtcatctataatttataagtgcaacattcactca

P-mucrosquamatus ------------------------------------------------------------

B-jararaca_I.3.1 ------------------------------------------------------------

B-jararaca_D.10 ------------------------------------------------------------

B-jararaca_D.07 ------------------------------------------------------------

B-atrox cagtgtgcataggagtctcaaggtgcagaatcagatttggaacaggataatgtatggttg

C-horridus ------------------------------------------------------------

C-viridis atatacatccatgtcaagaaagagatggattcatactatgtgcagagctttttaattgct

P-mucrosquamatus ------------------------------------------------------------

B-jararaca_I.3.1 ------------------------------------------------------------

B-jararaca_D.10 ------------------------------------------------------------

B-jararaca_D.07 ------------------------------------------------------------

B-atrox gatccgagagctcgagggttaataggaggagggttcagtaagtaaaaaaaggaaatgacg

C-horridus ------------------------------------------------------------

C-viridis aaacagcccacaggtgaattcatgcttgaagtttttgcataaacaatggaa---------

P-mucrosquamatus ------------------------------------------------------------

B-jararaca_I.3.1 ------------------------------------------------------------

B-jararaca_D.10 ------------------------------------------------------------

B-jararaca_D.07 ------------------------------------------------------------

B-atrox tatcggacactatagtagaggtgggaaggtgagtacgcccttattacgaggaacgtttat

C-horridus -------------------------------------------------------tttat

C-viridis -----------atagcttattcaatcagcttgaatacatttcttcagtgaacccatatct

P-mucrosquamatus -------------------------------------------------------tttat

B-jararaca_I.3.1 -------------------------------------------------------tttat

B-jararaca_D.10 -------------------------------------------------------tttat

B-jararaca_D.07 -------------------------------------------------------tttat

* * *

B-atrox caatgagagaattggaaacttttgcaaggctttgaaggtctgatgttttgaaaaacctga

C-horridus ctat--------------------------------------------------------

C-viridis ctaggacattgactgtatctcttcctatttctggcaggttttccccccaaacacagtagg

P-mucrosquamatus ctat--------------------------------------------------------

B-jararaca_I.3.1 ctat--------------------------------------------------------

B-jararaca_D.10 ctat--------------------------------------------------------

B-jararaca_D.07 ctat--------------------------------------------------------

* *

B-atrox cagcgaactataatgagatattggacaatggaatgaagaccgagtgagtaattggaaaat

C-horridus ------------------------------------------------------------

C-viridis agaaagtgggttanaaaaaaaaatcagtaaaacataaaacataaaaagatgatgaagtga

P-mucrosquamatus ------------------------------------------------------------

B-jararaca_I.3.1 ------------------------------------------------------------

B-jararaca_D.10 ------------------------------------------------------------

B-jararaca_D.07 ------------------------------------------------------------

B-atrox ttggcagaagtttgctgtaatctaaatgtaaataatgagtctttgatatttctgccagca

C-horridus ------------------------------------------------------------

C-viridis gaggcttctctctcccttaccaaaggcctaggtgaggcactacatcttaatatctcttct

P-mucrosquamatus ------------------------------------------------------------

B-jararaca_I.3.1 ------------------------------------------------------------

B-jararaca_D.10 ------------------------------------------------------------

B-jararaca_D.07 ------------------------------------------------------------

B-atrox aagtgtcagcagcttcttgttaaatgaatccttttgaaatgtatttaatacatcttttaa

C-horridus ------------------------------------------------------------

C-viridis aaacaagagcag-------------------------agtgggcaccatccagtttttgt

P-mucrosquamatus ------------------------------------------------------------

B-jararaca_I.3.1 ------------------------------------------------------------

B-jararaca_D.10 ------------------------------------------------------------

B-jararaca_D.07 ------------------------------------------------------------

B-atrox aactctctttagctgtttttaatgtgattgtgagcgccggacagaacacccaccattttt

C-horridus ------------------------------------------------cctaccatcctt

C-viridis gttcgtctttatctat--------------------------------cccaccatcctt

P-mucrosquamatus ------------------------------------------------tcaaccatcctt

B-jararaca_I.3.1 ------------------------------------------------cccaccatcctt

B-jararaca_D.10 ------------------------------------------------cccaccatcctt

B-jararaca_D.07 ------------------------------------------------cccaccatcctt

* ***** **

B-atrox tgagattgactgggttgagagaggatgagcgcaatacaaagtcataacattagtaccgta

C-horridus tgagataggctgggttgagagaggatgtgcacaattgaaagtcataacattagtattgta

C-viridis tcagataggctgggttgagagaggatgtgcgcaatacaaagtcataacattagtaccgtg

P-mucrosquamatus tgagataggctgggttgagagaggatgtgcgcaatacaaagtcataacattagtactgta

B-jararaca_I.3.1 tgagattgactgggttgagagaggatgtgcacaatacaaagtcataatattagtaccata

B-jararaca_D.10 tgagattgactgggttgagagaggatgtgcacaatacaaagtcataatattagtaccata

B-jararaca_D.07 tgagattgactgggttgagagaggatgtgcacaatacaaagtcataatattagtaccata

* **** * ****************** ** **** ********** ******* *

B-atrox accgcatgggttctatctttaata--gtcatctataatttataagtgcaacattcacaca

C-horridus accacatgggttctatctttaacat--tcatttataatttataagtgcaacattcacaca

C-viridis accacatgggttctatctttaacacagtcatctataatttataagtgcaacattcactca

P-mucrosquamatus accacatgggttctatctttaacacagtcatttataatttataactgcaacatttacaca

B-jararaca_I.3.1 accacatgggttctatctttaacacagacatctagaatttataagtgcaacattcacaca

B-jararaca_D.10 accacatgggttctatctttaacacagacatctagaatttataagtgcaacattcacaca

B-jararaca_D.07 accacatgggttctatctttaacacagacatctagaatttataagtgcaacattcacaca

*** ****************** * *** ** ********* ********* ** **

B-atrox atacacatccaagtcaagaaagagatggattcatactatatgctgagcttct--gatgct

C-horridus atacacatccatgtcaagaaagagatggattcatactatatgctgagctttctgaatact

C-viridis atatacatccatgtcaagaaagagatggattcatactatgtgcagagctttttaattgct

P-mucrosquamatus atacacatcccagtcaagaaagagatggattcatagtatatgccaagctttctgtgtgct

B-jararaca_I.3.1 atacacatccaagtcaagaaagagatggattcatactatatgctgagctttctcagtgct

B-jararaca_D.10 atacacatccaagtcaagaaagagatggattcatactatatgctgagctttctcagtgct

B-jararaca_D.07 atacacatccaagtcaagaaagagatggattcatactatatgctgagctttctcagtgct

*** ****** *********************** *** *** ***** * **

B-atrox aaacgg-atacaggtgaattcacgcttcaagattttgtataaac-atggaaatagcttat

C-horridus aagcagcatacaggtgaattcacac---------ttgcataaacaat-aaaatagcttat

C-viridis aaacagcccacaggtgaattcatgcttgaagtttttgcataaacaatggaaatagcttat

P-mucrosquamatus aaacagcacacaggtgaattcatgcttcaagattttgtataaacaatggaaatagcttat

B-jararaca_I.3.1 aaacagcacacaggtgaattcacgcttcaagattttgtataaacaatggaaatagcttat

B-jararaca_D.10 aaacagcacacaggtgaattcacgcttcaagattttgtataaacaatggaaatagcttat

B-jararaca_D.07 aaacagcacacaggtgaattcacgcttcaagattttgtataaacaatggaaatagcttat

** * * ************* * *** ****** ** ***********

B-atrox tcaatcaccttaaataaatttcttgagtgaacccagatctcttggacattgactgtaact

C-horridus tcaatcaccttgaataaatttcttgagtgaacccagatctctaggacattgactgtaact

C-viridis tcaatcagcttgaatacatttcttcagtgaacccatatctctaggacattgactgtatct

P-mucrosquamatus tcaatcaccttgaataaatttcttgagtgaacccagatctctaggacattgactgtaact

B-jararaca_I.3.1 tcaatcaccttaaataaatttcttgagtgaacccagatctctaggacattgactgtaact

B-jararaca_D.10 tcaatcaccttaaataaatttcttgagtgaacccagatctctaggacattgactgtaact

B-jararaca_D.07 tcaatcaccttaaataaatttcttgagtgaacccagatctctaggacattgactgtaact

******* *** **** ******* ********** ****** ************** **

B-atrox cttccaggtttctggcagttc--cccccccccaaacacaattggagaagttaggttatgg

C-horridus tttccaggtttcaggcaggtt---tttccctcaaacacagtaggagaaggtaacttatgg

C-viridis cttcc-tatttctggcaggtt---ttccccccaaacacagtaggagaaagtgggttatgg

P-mucrosquamatus cttccaggtttctggcaggtt---tttcccacaaacaccattggagaaggaaggttatgg

B-jararaca_I.3.1 cttccaggttttcggcaggttcccccccccccaaacacaattggagaagttaggttatgg

B-jararaca_D.10 cttccaggtttctggcaggttcccccccccccaaacacaattggagaagttaggttatgg

B-jararaca_D.07 cttccaggtttctggcaggttcccccccccccaaacacaattggagaagttaggttatgg

**** *** ***** * *** ******* * ****** ******

B-atrox ccctttcttgtttggcgctaacgtacctgcttacccg-gaaaatgaggtgaaggaccatc

C-horridus ccctttcttgtttggcacaaatgatcctgcttgagggcgaaaatgaggtcaaggaccatc

C-viridis ccctttcttgtttggtgctaacaatcttgcttgagggccaaaatgaggtcaaggaccatc

P-mucrosquamatus ccctttcatgtctggctctaacaatcctgcttgagggccaaaatgaggtcaaggaccaac

B-jararaca_I.3.1 ccctttcttgtttggcgctaacgatcctgcttgagggccaaaatgaggtgaaggaccatc

B-jararaca_D.10 ccctttcttgtttggcgctaacgatcctgcttgagggccaaaatgaggtgaaggaccatc

B-jararaca_D.07 ccctttcttgtttggcgctaacgatcctgcttgagggccaaaatgaggtgaaggaccatc

******* *** *** * ** * ***** * ********** ******** *

B-atrox ccaagtaatatagagttt-cgttaagttgttgttgttaa---cttgttgtttttcttgta

C-horridus ccaagtaacatagagtttactttaagttgttgttgttaa---cttgttgtttttcttgta

C-viridis ccaagtaatatagagtttactttaagttgttgttgttaa---cttcctgtttttcttgta

P-mucrosquamatus acaagtaatat--agtttactttaagttgttgttgttaacttcttgttgtttttcttgta

B-jararaca_I.3.1 ccaagtaatatagagtttactttaagttgttgttgttaa---cttgttgtttttcttgta

B-jararaca_D.10 ccaagtaatatagagtttactttaagttgttgttgttaa---cttgttgtttttcttgta

B-jararaca_D.07 ccaagtaatatagagtttactttaagttgttgttgttaa---cttgttgtttttcttgta

******* ** ***** * ****************** *** *************

Exon 3

B-atrox gAAGATTTATGCGCATACACCTTGGTAAACATGCCGGAAGTGTAGCAAATTATGATGAGG

C-horridus gGAAAAAAATGAAGTTACAGTTTGGTCTGCATAGCAAAAATGTACCAAACAAGGATAAGC

C-viridis gGAGAAATATCCGGATAAAGCTTGGTATGCATAGCAAAACTGTACCAAATGAGGATGAGC

P-mucrosquamatus gGAAAGATATGGAGATATACCTTGGTGTGCATAGCAAAAAGGTACCAAATAAGGATGTGC

B-jararaca_I.3.1 gGAGATTTATGCGCATATACCTTGGTATACATGCCCGAAGTGTAGCAAATGATGATGAGG

B-jararaca_D.10 gGAGATTTATGCGCATATACCTTGGTATACATGCCCGAAGTGTAGCAAATGATGATGAGG

B-jararaca_D.07 gGAGATTTATGCGCATATACCTTGGTATACATGCCCGAAGTGTAGCAAATGATGATGAGG

* * * ** ** * ***** *** * ** *** **** * *** *

Cys

B-atrox TGGTAAGATACCCAAAGGAGAAGTTCATT**TGT**CCCAATAAGAAAAAAAATGTCATAACGG

C-horridus AGACAAGAGTCCCAAAGGAGAAGTTCTTT**TGT**CTTAGTAGCAAAAACAATAAAGAATGGG

C-viridis AGACAAGAGTCCCAAAGGAGAAGTTCTTT**TGT**CTTAGTAACAAAAACTATACCCTTTGGG

P-mucrosquamatus AGAGAAGAGTCCCAAAGGAGAAGTTCTTT**TGT**GACAGTAGCAAAACCTACACCAAATGGA

B-jararaca_I.3.1 TGATAAGATACCCAAAGGAGAAGTTCATT**TGT**CCCAATAAGAATATGAGTGACGAAAAGG

B-jararaca_D.10 TGATAAGATACCCAAAGGAGAAGTTCATT**TGT**CCCAATAAGAATATGAGTGACGAAAAGG

B-jararaca_D.07 TGATAAGATACCCAAAGGAGAAGTTCATT**TGT**CCCAATAAGAATATGAGTGACGAAAAGG

* **** **************** ***** * ** ** * *

Asp

B-atrox ACAAG**GAC**ATTATGTTGATCAGGCTGGACAGACCTGTCAAAAACAGTGAACACATCGCGC

C-horridus ACAAG**GAC**ATCATGTTGATCAGGCTGAACCGTCCTGTTAACAACAGTAAACACATTGCGC

C-viridis ACAAG**GAC**ATCATGTTGATCAGGCTGGACAGCCCTGTTAGCAACAGTGAACATATCGCGC

P-mucrosquamatus ACAAG**GAC**ATCATGTTGATCAGGCTGGACAGACCTGTCAGGAAGAGTGCACACATCGCGC

B-jararaca_I.3.1 ACAAG**GAC**ATTATGTTGATCAGGCTGAACAGACCTGTCAAAAACAGCACACACATCGCAC

B-jararaca_D.10 ACAAG**GAC**ATTATGTTGATCAGGCTGCCCAGACCTGTCAAAAACAGCACACACATCGCAC

B-jararaca_D.07 ACAAG**GAC**ATTATGTTGATCAGGCTGAACAGAC--GTCAAAAACAGCACACACATCGCAC

********** *************** * * * ** * ** ** *** ** ** *

Cys

B-atrox CTCTCAGCTTGCCTTCCAACCCTCCCAGTGTGGGCTCAGTT**TGC**CGTATTATGGGAT-GG

C-horridus CTCTCAGCTTGCCTTCCAAGCCTCCCAGTCAGGACACAGTT**TGC**AATATTATGGGAT-GG

C-viridis CACTCAGCTTGCCTTCCAGCCCTCCCAGTGTGGGCTCAGTT**TGC**CGTATTATGGGAT-GG

P-mucrosquamatus CTCTCAGCTTGCCTTCCAGCCCTCCCAGTGTGGGCTCAGTT**TGC**CGTGTTATGGGAT-GG

B-jararaca_I.3.1 CTATCAGCTTGCCTTCCAACCCTCCCAGTGTGGGCTCAGTT**TGC**CGTGTTATGGGAT-GG

B-jararaca_D.10 CTATCAGCTTGCCTTCCAACCCTCCTAGTGTGGGCTCAGTT**TGC**CGTGTTATGGGATGGG

B-jararaca_D.07 CTATCAGCTTGCCTTCCAACCCTCCTAGTGTGGGCTCAGTT**TGC**CGTGTTATGGGAT-GG

* *************** ***** *** ** * ******** * ********* **

Intron 3

B-atrox GGCGCAATCACAACTTCTGAAGGTAGGGATCTCTgtctata--ctcagacccagctttga

C-horridus GGCACAATCTCACCTACTAAAGGTAGGGATCTCTgtctatattctcagacc---------

C-viridis GGCAGAATCTCACCTAGTAAAGgtagggatctctatctatactctcagactcagcttttg

P-mucrosquamatus GGCACAATCACATCTCCTCAAGGTAGGGATCTCTgtctatactctcagacccagcttttg

B-jararaca_I.3.1 GGCTCAATCACAATTCCTAACGGTAGGGATCTCTgtctatactctcagacccagcttttg

B-jararaca_D.10 GGCTCAATCACAATTCCTAACGGTAGGGATCTCTgtctatactctcagacccagcttttg

B-jararaca_D.07 GGCTCAATCACAATTCCTAACGGTAGGGATCTCTgtctatactctcagacccagcttttg

*** **** ** * * * ************* ****** *******

B-atrox t--taacatcaatcaggtatagggtcccactatgtcacccatgaat-attggaatgtggg

C-horridus ---------caactaggtctagggtcccactatgtcacccatgaat-attggaatgtgga

C-viridis atataacatcagctaggtctagggtcccactatgtcacccatgaat-attggaatgtgga

P-mucrosquamatus atgtaacatcaactaggtctagggtcccactatgtcacccatgaat-attggaatgtgga

B-jararaca_I.3.1 ttgtaacatcaactaggtctggggtcccactatgtcacccatgaataattggaatgtgga

B-jararaca_D.10 ttgtaacatcaactaggtctggggtcccactatgtcacccatgaat-attggaatgtgga

B-jararaca_D.07 ttgtaacatcaactaggtctggggtcccactatgtcacccatgaat-attggaatgtgga

** **** * ************************* ************

B-atrox tggacaatgggattccctaagattccatttcttcatcagttccacacccacaaggtcctt

C-horridus tggacaatgggattccctaagattccatttcttcatcagttccacacccacaaggtcctt

C-viridis tggacaatgggattccctaagattccatttcttcatccgttccacacccacaaggtcctt

P-mucrosquamatus tggacaatgggattccctaagattccatttcttcatcagttccacacccacaaggtcctt

B-jararaca_I.3.1 tggacaatgggattccctaagattccatttcttcatcagttctacgaccacaaggtcctt

B-jararaca_D.10 tggacaatgggattccctaagattccatttcttcatcagttctacgaccacaaggtcctt

B-jararaca_D.07 tggacaatgggattccctaagattccatttcttcatcagttctacgaccacaaggtcctt

************************************* **** ** *************

B-atrox ggaatgaaagaaataagtaaggaactgtcatactgtatagctaaaaaaaa--cctatagg

C-horridus ggaataaaataaataagtaaggaactggcatgctgtatagctgaaaaaaaaccctatagg

C-viridis ggaataaaagaaataagtaaggaactggcatgctgtatagctaaaaaaaa-ccctatagg

P-mucrosquamatus ggaataaaataaataagtaaggaagtggcatgctgtgtagctaaaaaaaaaccctatagg

B-jararaca_I.3.1 ggaatgaaagaaataagtaaggaactgtcatgctgtatagctaaaaaaaaa-cctatagg

B-jararaca_D.10 ggaatgaaagaaataagtaaggaactgtcatgctgtatagcttaaaaaaaa-cctatagg

B-jararaca_D.07 ggaatgaaagaaataagtaaggaactgtcatgctgtatagcttaaaaaaaa-cctatagg

***** *** ************** ** *** **** ***** ******* ********

B-atrox atca-gggctttttatagcagagctgttgggaatcagaaaaa-accattagcatggcagt

C-horridus atca-ggtttatttatagcagtgctgttgggaatcagaaaagtcccatta-catggcagt

C-viridis atca-ggtttatttatagcagtgctgttgggaatcagaaaagtcccattagcatggcagt

P-mucrosquamatus atca-ggtttacttatagcagtgctgttgggaatcagaaaagtcccattagcatggcagt

B-jararaca_I.3.1 atcagggtttttttatagcagagctgttgggaatcagaaaagtcccattagcatggcagt

B-jararaca_D.10 atcagggtttttttatagcagagctgttgggaatcagaaaagtcccattagcatggcagt

B-jararaca_D.07 atcagggtttttttatagcagagctgttgggaatcagaaaagtcccattagcatggcagt

**** ** * ********* ******************* ****** *********

B-atrox cttgcctgacttaattacctttaataacgg---gtgttttgaatcgatgcgaac------

C-horridus cttgcctggcttcattacctttaataatggggtgttttgtgaatggccatggccattttg

C-viridis cttgccaggcttcattacctttaataatgggatgttttgtgaaaggccatggcccttttg

P-mucrosquamatus cttgcctggcttcattacctttaataatggggtgttttgtgaatggccatggccgttttg

B-jararaca_I.3.1 cttccctggtttaattacctttaataacggggtgttttctgaatggccatggccgtttca

B-jararaca_D.10 cttccctgg---------ctttaataacagggtgttttctgaatggccatggccgtttca

B-jararaca_D.07 cttccctgg---------ctttaataacagggtgttttctgaatggccatggccgtttca

*** ** * ********* * ** ** **** * * *

B-atrox -agaatgagaagtctccagcttaggaagttttcagagtatatttaccacttgctttcaaa

C-horridus gaaacggagaagtctcccgcagatgatgttttcagagtatatttatctcctgctctcaaa

C-viridis gaaatggagaagtctcctgcggaggatgttttcagagtacatttatctcttgctctcaaa

P-mucrosquamatus gaaatgaagaagtgtcccgcggaggatgttttcagagtatatttaccccttgctctcaaa

B-jararaca_I.3.1 gaaatggagaagtctcctgcttaggaagttttcagagtatatttaccacttgctctcaaa

B-jararaca_D.10 gaaatggagaagtctcctgcttaggaagttttcagagtatatttaccacttgctctcaaa

B-jararaca_D.07 gaaatggagaagtctcctgcttaggaagttttcagagtatatttaccacttgctctcaaa

* * ****** *** ** * ** ************ ***** * * **** *****

B-atrox atgccaactatgaactcaaaatcatcctctatccttggatggatataaa-tctctgtgga

C-horridus ataccaaatatgaactcaaaaacgtggtctatccttggatggatataaactctctgtgga

C-viridis ataccaaatatgaactcaaaaacatggtctatccttggatggatattaaatatctgtgga

P-mucrosquamatus ataccaaatatgaactcaaaaacatggtctatccttggatggatataaaatctctgtgga

B-jararaca_I.3.1 ataccaactatgaactcaaaaacatggtttatccttggatggatataaaatctctgtgga

B-jararaca_D.10 ataccaactatgaactcaaaaacatggtttatccttggatggatataaaatctctgtgga

B-jararaca_D.07 ataccaactatgaactcaaaaacatggtttatccttggatggatataaaatctctgtgga

** **** ************* * * * ***************** ** * ********

B-atrox tggttgcagtggacgtgttccttttaagccctggtggtgcagtggtcagaatgcagtatt

C-horridus tggttgcagaggatttcttccttctaagtcctggtggtgcagtggttagaatgcagtatt

C-viridis tgattgcagatgatttcttccttttaagtcctggtggtgcagtggttagaatgcagtatt

P-mucrosquamatus tggttgcagaggacttcttccttttaagtcctggtggtgcagtggttagaatgcagtatt

B-jararaca_I.3.1 tggttgcagaggacgtcttccttctaagtcctggtggtgcagtggttagaatgcagtatt

B-jararaca_D.10 tggttgcagaggacgtcttccttctaagtcctggtggtgcagtggttagaatgcagtatt

B-jararaca_D.07 tggttgcagaggacgtcttccttctaagtcctggtggtgcagtggttagaatgcagtatt

** ****** ** * ****** **** ***************** *************

B-atrox gcaggctatctctgctcccagcctggaatttgatcctgatggggctcaaggttgacccca

C-horridus gcaggctatctctgctcctaacctggaattcaatcctggcggggctcaaggttga-ctca

C-viridis gcaggctatctctgctcccagcctggaattcgatcctgacggggctcaaggttga-ctca

P-mucrosquamatus gctggctatctctgctcccagcctggaattcgatcctgacagagctcaaggttga-ctca

B-jararaca_I.3.1 gctggctatctctgctcccagcctggaattcgatcctgatggggctcaaggttga-ccca

B-jararaca_D.10 gctggctatctctgctcccagcctggaattcgatcctgacggggctcaaggttga-ccca

B-jararaca_D.07 gctggctatctctgctcccagcctggaattcgatcctgacggggctcaaggttga-ccca

** *************** * ********* ****** * ************ * **

B-atrox gccttccagccttttgaggttgacaagaggaggactcagactgttgggggcaatatgtgg

C-horridus gccttccatccttttgaggttgataaaaggaggactcagattgttaggggcaatatgctg

C-viridis gccttccatccttttgaggttgataaaaggaggactcagactgttgggggcaatatgctg

P-mucrosquamatus gccttccatccttttgaggttgataaaaggaggactcagattgttgggggcaatttgctg

B-jararaca_I.3.1 gccttccatccttttgaggttgataaaaggaggactcagactgttgggggcaatatgtgg

B-jararaca_D.10 gccttccatccttttgaggttgataagaggaggactcagactgttgggggcaatatgtgg

B-jararaca_D.07 gccttccatccttttgaggttgataagaggaggactcagactgttgggggcaatatgtgg

******** ************** ** ************* **** ******** ** *

B-atrox atgctgtaaatcaccaagaga--gtgcagtgcataggtctaag------tgctattgcta

C-horridus atattgtaaatcacccagagagtgtgcagtatataagtctaag------tgctattgcta

C-viridis atattgtaaatcacccagaga--gtgcagtatataggtctaagtactattgctattgcta

P-mucrosquamatus atattgtaaatcacccagaga--gtgcagtatataggtctaagtgctattgctattgcta

B-jararaca_I.3.1 atgctgtaaatcaccaaaaga--gtgcagtatataggtctaag------tgctattgcta

B-jararaca_D.10 atgctgtaaatcaccaaaaga--gtgcagtatataggtctaag------tgctattgcta

B-jararaca_D.07 atgctgtaaatcaccaaaaga--gtgcagtatataggtctaag------tgctattgcta

** *********** * *** ******* *** ******* ***********

B-atrox gtaaggccatggaagagttacatcgattctcagctaaggcttatggtcatccacacattc

C-horridus gtaaggccatggaagtgttacatgcactctcagctttaccttatggtcattcacacattc

C-viridis gtaagg-catgtaagtgttatgtgtgctctcggctttggcttatggtcatccacacattc

P-mucrosquamatus gtaaggccatggaagtgttacatgcactctaagctttgacttatggtcatccacacattc

B-jararaca_I.3.1 gtaaggccatggaagtgttacatgcactctcagctttggcttatggtcatc--cacattc

B-jararaca_D.10 gtaaggccatggaagtgttacatgcactctcagctttggcttatggtcatccacacattc

B-jararaca_D.07 gtaaggccatggaagtgttacatgcactctcagctttggcttat----------------

****** **** *** **** * *** *** *****

B-atrox aagcctgtcaagatacctagatcttgtaagcccaccttgttttctacctaggtttctacc

C-horridus aagcctgtcaaggtaggtagatcttgtaagcccatcttgttttctacttaggttattatc

C-viridis aagcttgtcaaggtaggtagatcttgtaagcctaccttgttttctacctagatttctacc

P-mucrosquamatus aagcctgccaaggtaggtagatcttgtaagcctaccgtgttttctacgtaggtttctacc

B-jararaca_I.3.1 aagcctgccaaggtaggtagatcttgtaagcccaccttgttttctacctaggtttctacc

B-jararaca_D.10 aagcctgccaaggtaggtagatcttgtaagcccaccttgttttctacctaggtttctacc

B-jararaca_D.07 ------------------------------------------------------------

B-atrox agataggtgtggctagagcagaaaagtcacacagccttggtcaagagctgggaaataatt

C-horridus aaatagatgtagctagagcagacatgtcacacagccttggtcaagagctgggaaataatt

C-viridis agataggtgtagttagagcagacaagtcacacagccttggtcaagagctgggaaataatt

P-mucrosquamatus aaataggtatagctagagcagacaagtcacacagccttggtcaagagctgggaaataatt

B-jararaca_I.3.1 agataggtgtggctagagcagaaaagtcacacagccttggtcaagagctgggaaataatt

B-jararaca_D.10 agataggtgtggctagagcagaaaagtcacacagccttggtcaagagctgggaaataatt

B-jararaca_D.07 ------------------------------------------------------------

B-atrox aaggagttacgcaaataactttcaactaaccactgaagcactatagtttttttggaatac

C-horridus aaggactcacaaaaataattttcaattaaacactgagacaccacagtttttttggaatac

C-viridis aaggactcacacaaataattttcaactaactactgaggcacctcaggatttttggaatac

P-mucrosquamatus aaggactcacacaaaaaattttcaactaaccactgaggcaccacaagtttttgggaatac

B-jararaca_I.3.1 aaggactcacacaaataattttcaactaaccactgaagcactatagtttttttggaatac

B-jararaca_D.10 aaggactcacacaaataattttcaactaaccactgaagcactatagtttttttggaatac

B-jararaca_D.07 -------------------tttcaactaaccactgaagcactatagtttttttggaatac

****** *** ***** *** * **** *******

B-atrox atggagaccccgtgaattt-aagtaacagc-aaacattactgcttgcttagtaaattta

C-horridus atggagatccagtgaatttaaaataacagcaaaactttactgctcgcaatgtaaatttc

C-viridis atggagacccagtgaatttaaagtaacatcaaaactttactgcttgcgatgtaaatttg

P-mucrosquamatus acggagacccagtgaatttaaagtaacagcaaaactttactgcttgcgatataaatttg

B-jararaca_I.3.1 atggagaccccgtgaatttaaagtaacaccaaaacattactgcttgcaatgtaaattta

B-jararaca_D.10 atggagaccccgtgaatttaaagtaacaccaaaacattactgcttgcaatgtaaattta

B-jararaca_D.07 atggagaccccgtgaatttaaagtaacaccaaaacattactgcttgcaatgtaaattta

* ***** ** ******** ** ***** * **** ******** ** *******

B-atrox ttctttctcatatgcacacatgaagggatggatttttagatacattcttctcagttaggc

C-horridus ctctttctcatacccacacatgcacccatccatatttagacacatccttctcaattaggc

C-viridis ctctttctcatacccacacatgcacccatccatgtttagacacatccttctcaattaggc

P-mucrosquamatus ctctttctcatatgcacacatgcacccatccatgtttagacacatccttatcaattaggc

B-jararaca_I.3.1 ttc--tctcatatgcacacatgaacccatccatttttagatatattcttctcagttaggc

B-jararaca_D.10 ttc--tctcatatgcacacatgaacccatccatttttagatatattcttctcagttaggc

B-jararaca_D.07 ttc--tctcatatgcacacatgaacccatccatttttagatatattcttctcagttaggc

** ******* ******** * ** ** ****** * ** *** *** ******

B-atrox tgcc-------------agggacagaaactcctaagttctggatccaaagatcactcaag

C-horridus tgccaggcaccaactagtgggacagaaactcctaagttgtggatccaaa-atgactcaag

C-viridis tgccaggcattagctagagggacagaaactcctaagttgtggatgcaaagatgactcaag

P-mucrosquamatus tgccaggcaccagctagagggacagaaactcctaagttgtggatccaaagatgactcaag

B-jararaca_I.3.1 tgcc-------------agggacagaaactcctaagttgtggatccaaagatgactcaag

B-jararaca_D.10 tgcc-------------agggacagaaactcctaagttgtggatccaaagatgactcaag

B-jararaca_D.07 tgcc-------------agggacagaaactcctaagttgtggatccaaagatgactcaag

**** ******************** ***** **** ** *******

B-atrox gagtgggtcagctttaggacccctttttatcctcaaaggttcttgatttgttgattagct

C-horridus gagttggtgagctttaggacccccttttatcctgaaaagttcttgatttgttgattagct

C-viridis gagtgggtgaactttaggacctctttttatcctgaaaagttcttgatttgttgattagct

P-mucrosquamatus gagtgggtgagctttaggatccctttttatactgaaaatgtcttgatttgttgattagct

B-jararaca_I.3.1 gagtgggtgagctttaggacccctttttatcctcaaaggttcttgatttgttgattacct

B-jararaca_D.10 gagtgggtgagctttaggacccctttttatcctcaaaggttcttgatttgttgattacct

B-jararaca_D.07 gagtgggtgagctttaggacccctttttatcctcaaaggttcttgatttgttgattacct

**** *** * ******** * * ****** ** *** ***************** **

B-atrox tgaattggggtttatttcgtattctcaggctcaactttgtccgattcagatgacatttt-

C-horridus gggattggggtttattccatattctcaggctcagctttgtctgattcagatgactttttt

C-viridis gggattggggtttattccatattctcaggctcaactttgtctgattcagatgacattttt

P-mucrosquamatus gggattggggtttattccatattctcaggctcaactttgtttgattcaaatgacattttg

B-jararaca_I.3.1 agaattggggtttatttcatattctcaggctcaactttgtctgattcagatgacatttt-

B-jararaca_D.10 agaattggggtttattttatattctcaggctcaactttgtctgattcagatgacatttt-

B-jararaca_D.07 agaattggggtttattttatattctcaggctcaactttgtctgattcagatgacatttt-

* ************* ************** ****** ****** ***** ****

B-atrox -ccctgtttccttagacttatttccacctcatctgtcc-tctccaagggatcttttggaa

C-horridus cctttgtttccttagacttatttccccctcatctgtccttccccaggggatcttttagac

C-viridis cttttgtttccttacacttatttccatctcatctgtcc-tccccaagggatcttttggac

P-mucrosquamatus cctttttttccttacacttatttccatctcatctgtcc-tccgcaagggatcttttggac

B-jararaca_I.3.1 -ctctgtttccttagacttatttccacctcatctgtcc-tccccaagggatcttttggac

B-jararaca_D.10 -ctctgtttccttagacttatttccacctcatctgtcc-tccccaagggatcttttggac

B-jararaca_D.07 -ctctgtttccttagacttatttccacctcatctgtcc-tccccaagggatcttttggac

* ******** ********** *********** ** ** ********** **

B-atrox aagattccttg-cttttt-gtcttatagagaatacaaacttacaggtctgcttcatcttc

C-horridus aagattcctgg-ctttttgttcttgtacacaatagaaacttatgggtctgcttcatcttc

C-viridis aagattcctgg-ctttttgttcttgtacacaatacaaacctatgcgtcttcttcatcttc

P-mucrosquamatus aagattcctgatttttttgttcttgtacacaatacaaacttatgggtctgcttcatcttc

B-jararaca_I.3.1 aagattcctgg-cttttt-gtcttatacacaatacaaacttacaggtctgcttcatcttc

B-jararaca_D.10 aagattcctgg-cttttt-gtcttatacacaatacaaacttacaggtctgcttcatcttc

B-jararaca_D.07 aagattcctgg-cttttt-gtcttatacacaatacaaacttacaggtctgcttcatcttc

********* ***** **** ** * **** **** ** **** **********

B-atrox ggatatggaagtttcagtatgctggctgaagaattttgcaagtggaagttcacaagtctt

C-horridus agatatggaagtttcagtatgctggctgaggaattttgggagtggaagttcacaagtctt

C-viridis agatatggaagtttcagtattctggctgaggaattttgggagtgcaagttcccaagtctt

P-mucrosquamatus agatatggaagtttcgatatgcaggctgaggaattttgggagtggaagttcacaagtctt

B-jararaca_I.3.1 agatatggaagtttcagtatgctggctgaagaattttgcgagtggaagttcacaagtctt

B-jararaca_D.10 agatatggaagtttcagtatgctggctgaagaattttgcgagtggaaattcacaagtctt

B-jararaca_D.07 agatatggaagtttcagtatgctggctgaagaattttgcgagtggaaattcacaagtctt

************** *** * ****** ******** **** ** *** ********

B-atrox aaagttgccaagtttggaaacccctgttctaggtcatctccaggggtacgaagatcagtt

C-horridus aaagttgccaagtttggagaccgccgttctaggtcatctccaggggatgcgagatcagtt

C-viridis aaagtggccaagtttggagacccccattctaggtcatctccaggggatgcgagatcagtt

P-mucrosquamatus aaagttgccaagtttggaaacccccgttctaggtcatctccacgggatgcgagatcagtt

B-jararaca_I.3.1 aaagttgccaagttgggagacccctgttctagttcatctccaggggatgcaagatcagtt

B-jararaca_D.10 aaagttgccaagttgggagacccctgttctaggtcatctccaggggatgcaagatcagtt

B-jararaca_D.07 aaagttgccaagttgggagacccctgttctaggtcatctccaggggatgcaagatcagtt

***** ******** *** *** * ****** ********* *** *********

B-atrox ctgtatcttttgaactacgaaaagccccacttttccaacaacggcaatctttacgtagat

C-horridus ctgtgacttttgaactagcaaaagaatcacttgtccaacaa-ggcaatctttgcatagag

C-viridis ctgtgacttttgaactagcaaaaa-cccacttttccaacaa-ggcaatctttgcatagag

P-mucrosquamatus ctgtgacttttgaactagcaaaagccccacttttccaacaa-gggaatctttgcgtagag

B-jararaca_I.3.1 ctgtgacttttgaactagaaaaagccccacttttccaacaa-ggcaatctttacgtagag

B-jararaca_D.10 ctgtgacttttgaactagaaaaagccccacttttccaacaa-ggcaatctttacgtagag

B-jararaca_D.07 ctgtgacttttgaactag-aaaagccccacttttccaacaa-ggcaatctttacgtagag

**** *********** **** ***** ******** ** ******* * ****

B-atrox gtccg---------gagaagccctcaaatgcccatcagttgatttaagacctattaatat

C-horridus tgctggagaaatttgagaagccttcaaatgcccatcagctgatttaagatctattaatat

C-viridis tgctggagaaatttgagaaaccttcaaatgcccatcagctgatttaagacctactaatat

P-mucrosquamatus tgccggagaaatttgagaagccttcaaatgcccatcagctgatttaagacctattgatat

B-jararaca_I.3.1 tgccg---------gagaagccctcaattgcccatcagctgatttaagacctattaatat

B-jararaca_D.10 tgctg---------gagaagccctcaaatgcccatcagctgatttaagacctattaatat

B-jararaca_D.07 tgctg---------gagaagccctcaaatgcccatcagctgatttaagacctattaatat

* * ***** ** **** ********** ********** *** * ****

B-atrox cgtgacaaaaaacatcctgtgctatcgtgacaaaaaacatcctgtgcaaactttaactaa

C-horridus cttgacaaaaaacatcctgtgc------------aaaca------------tttaagtaa

C-viridis cttgacaaa-------------------------aaaca------------tttaactaa

P-mucrosquamatus cttgagaaa-------------------------aaaca------------tttaactaa

B-jararaca_I.3.1 ggtgacaaaaaacatcctgtgc------------aaaca------------tttaagtaa

B-jararaca_D.10 cgtgacaaaaaacatcctgtgc------------aaaca------------tttaagtaa

B-jararaca_D.07 cgtgacaaaaaacatcctgtgc------------aaaca------------tttaagtaa

*** *** ***** ***** ***

B-atrox ctctgtttttcccaggagtccctttgaacacctttgaaagacaatgcttcctatt-ccag

C-horridus atctgtttttcccaggagtccctttgaacaactttgaaagacaatacttcctatt-ccag

C-viridis atctgtttttcccaggtatccctttgaatggctttgaaagacaatacttcctattcccag

P-mucrosquamatus ctctttttttcccaggagtccctttgaacgtctttgaaaaacaatacttcttattcccag

B-jararaca_I.3.1 atctgttttttccaggagtccctttgaacagctttgaaagacaatacttcctatt-ccag

B-jararaca_D.10 atctgtttttcccaggagtccctttgaacagctttgaaagacaatacttcctatt-ccag

B-jararaca_D.07 atctgtttttcccaggagtccctttgaacagctttgaaagacaatacttcctatt-ccag

*** ***** ***** ********** ******** ***** **** **** ****

B-atrox gcaaggagatcagttcccttggttggagacaatggaaattggttggagacaatggaaatt

C-horridus gcaatgagatcagctcc--------------------gtttgttggagacaatggaaatt

C-viridis gcaatgagatcagctcc--------------------actggttggagacaatggaaatt

P-mucrosquamatus gcaatgagatcagctcc--------------------gttggttggagacaatggaaatt

B-jararaca_I.3.1 gcaaggagatcagttcc--------------------cttggttggagacaatggaaatt

B-jararaca_D.10 gcaaggagatcagttcc--------------------cttggttggagacaatggaaatt

B-jararaca_D.07 gcaaggagatcagttcc--------------------cttggttggagacaatggaaatt

**** ******** *** * *******************

B-atrox atagctctatgcatctaagtggtaaattgtgaaaggtaaaattttaatt-ttttaaaatt

C-horridus gtagctctatgcatctaggtggtaaattgtgaaagttagaattttgcctatttttta-tt

C-viridis atagctctatttatctaggtggtaaattgtgaaaggtagaattttgtctatttttta-tt

P-mucrosquamatus atagctctatgcatctaggtagtaaattgtgaaaggtagaattttgcctatgttttattt

B-jararaca_I.3.1 atagctctatgcatctaagtggtaaattgtgaaaggtacaatttt-cctttttttaaatt

B-jararaca_D.10 atagctctatacatctaagtggtaaatcgtgaaaggtacaatttt-ccttttttaaaatt

B-jararaca_D.07 atagctctatacatctaagtggtaaatcgtgaaaggtacaatttt-ccttttttaaaatt

********* ***** ** ****** ******* ** ****** * * ** * **

Exon 4 Cys

B-atrox cttttcagACACTTATCCCGATGTCCCTCAT**TGT**GCTAACATTAACCTGTTCAATAATAC

C-horridus tttttcagAGACTTATCCTGATGTCCCTCAT**TGT**GCTAACATTAACATACTCGATCATGT

C-viridis tttttcagAGACTTATCCCGATGTCCCTCAT**TGT**GCTAACATTAACCTACTCGATTATGA

P-mucrosquamatus ttttccagAGACTTATCCCGATGTCCCTCAT**TGT**GCTAACATTAACCTACTCGATTATGA

B-jararaca_I.3.1 cttttcagACACTTATCCCGATGTCCCTCAT**TGT**GCTAACATTAACCTGGTCAATGATAC

B-jararaca_D.10 cttttcagACACTTATCCCGATGTCCCTCAT**TGT**GCTAACATTAACCTGGTCAATGATAC

B-jararaca_D.07 cttttcagACACTTATCCCGATGTCCCTCAT**TGT**G--AACATTAACCTGGTCAATGATAC

*** **** ******** **************** ********* * ** ** **

Cys Cys

B-atrox GGTG**TGT**CGTGAAGCTTACAAT------GGGTTGCCGGCGAA------AACATTG**TGT**GC

C-horridus GGTT**TGT**CGAGCAATTTACCCA------GGGTTGCTGGAGAAAAGCAGAGTATTG**TGT**GC

C-viridis GGTG**TGT**CTAGCAGCTTACCCAGAATTTGGGTTGCCAGCGACAAGCAAAACATTG**TGT**GC

P-mucrosquamatus GGTA**TGT**CGAGCAGCTTACGCA------GGGTTGCCAGCAACAAGCAGAACATTG**TGC**GC

B-jararaca_I.3.1 GGTG**TGT**CGTGGAGCTTACAAG------AGGTTTCCGGCGAAAAGCAGAACATTG**TGT**GC

B-jararaca_D.10 GGTG**TGT**CGTGAAGCTTACAAT------GGGTTGCTGGGGAA------AACATTG**TGT**GC

B-jararaca_D.07 GGTG**TGT**CGTGAAGCTTACAAT------GGGTTGCTGGCGAA------AACATTG**TGT**GC

*** **** * * **** **** * * * * ****** **

Cys Intron 4

B-atrox AGGTGTCCTGCAAGGAGGCATAGATACA**TGT**GGGgtaagatgatccattttaagacagac

C-horridus AGGTATCCTGGAAGGAGGCAAAGATACA**TGT**GGAgtaagatgatccattttaagacagac

C-viridis AGGTATCCTGGAAGGAGGCAAAGATTCA**TGT**AAGgtaagatgatccgttttaagacagac

P-mucrosquamatus AGGTATCCTGGAAGGAGGCAAAGATTCA**TGT**GTGgtaagatgatccgttttaagacagac

B-jararaca_I.3.1 AGGTGTCCTGCAAGGAGGCAAAGATACA**TGT**GTGgtaagatgatccattttaagacagac

B-jararaca_D.10 AGGTGTCCTGCAAGGAGGCATAGATACA**TGT**GGGgtaagatgatccattttaagacagac

B-jararaca_D.07 AGGTGTCCTGCAAGGAGGCATAGATACA**TGT**GGGgtaagatgatccattttaagacagac

**** ***** ********* **** ***** ************ *************

B-atrox tcctaaagaaatgtagccaattttcaacgatatgatagagatctacccttgaaggcatct

C-horridus tcctaaaaaaatgatgctaattttcaaagctatgacagagatctagccttaaaggcatct

C-viridis tcctaaagaaatgatgccaaatttcaaagctgtgacagagatctagccttgaaggcatct

P-mucrosquamatus tcctaaagaaacgatgccaattttcaaagctatgacagagatctagccttgaaggcatct

B-jararaca_I.3.1 tcctaaagaaatgatgccaattttcaaagctatgatagagatctacccttgaagacatct

B-jararaca_D.10 tcctaaagaaatgatgccaattttcaaagctatgatggagatctacccttgaaggcatct

B-jararaca_D.07 tcctaaagaaatgatgccaattttcaaagctatgatggagatctacccttgaaggcatct

******* *** * ** ** ****** * * *** ******** **** *** *****

B-atrox tgtagaatcatacaaagttacggatttttctaactcaagtgccttctaggtgtttagatt

C-horridus tttagaaacatacaaagttatggctttttctaactcaagtgccttctaggtgtctagatt

C-viridis tgtagaatcatacaaagttacagctttttctaactcaagtgccttctaggtgtctagatt

P-mucrosquamatus tgtagaaacatacaaagttacgactctttctaactcaagtgccttctaggtgtctagatt

B-jararaca_I.3.1 tgtagaattatacaaagttacggctttttctaattcaggtgccttctaggtgtttagatt

B-jararaca_D.10 tgtagaattatacaaagttacggctttttctaactcaggtgccttctaggtgtttagatt

B-jararaca_D.07 tgtagaattatacaaagttacggctttttctaactcaggtgccttctaggtgtttagatt

* ***** *********** * ******* *** *************** ******

B-atrox tccagacttcccaatactggtgattctcatgggggaaactcaaggag-taaattcttcta

C-horridus tccagacttcccaatattggtaattttggtggagaaaa-tcaaggagttaaattcttcta

C-viridis tccagacttcccaatactggtgattctggtgggggaaactcaaggagttaaattcttcta

P-mucrosquamatus tccagacttcccaatattggtgattctggtgggggaaatacaaggagttaaattcttcta

B-jararaca_I.3.1 tccagacttcccaatactggtgattctcgtgggggaaactcaaggagttaaattcttcta

B-jararaca_D.10 tccagacttcccaatactggtgattctcgtgggggaaactcaaggagttaaattcttcta

B-jararaca_D.07 tccagacttcccaatactggtgattctcgtgggggaaactcaaggagttaaattcttcta

**************** **** *** * *** * *** ******* ************

B-atrox caaaggtcctagattgggcaacatggaagtgaaaca-ttgggggctataattgaatggat

C-horridus caaggatcctggattggggaacatggaagagaaaca-ttgggggctatatgtgaatggac

C-viridis caaggatcctggattggggaacatcgacgtgaaaca-ttgggggctatatgtgaatggac

P-mucrosquamatus caaggatcctggattggggaacatcgaagtgaaacatttgggggctatatgtgaatggac

B-jararaca_I.3.1 caaagatcctagattggacaacatggaagtgaaaca-ttgggggctatatgtgaatggac

B-jararaca_D.10 caaagatcctggattggggaacatggaagtgaaaca-ttgggggctatatgtgaatggac

B-jararaca_D.07 caaagatcctggattggggaacatggaagtgaaaca-ttgggggctatatgtgaatggac

*** * **** ****** ***** ** * ****** ************ ********

B-atrox gatgggaaggaattgtttgctccactgaacattcatagatacggtagtgatgtttcatgt

C-horridus aatgggagggaattgtttgctccactttccattcatagatatggtagtaatgtttcatgt

C-viridis gatgggagggaattgtttgctccccttccctttcatagatatggtagtaatgtttcatgt

P-mucrosquamatus gatgggagggaattgtttgctccactttccattcatagatacggtagtaaagtttcatgt

B-jararaca_I.3.1 gatgggaaggaattgtttgctccacttcccattcatagatacggtagtaatgtttcatgt

B-jararaca_D.10 gatgggaaggaattgtttgctccacttcccattcatagatacggtagtaatgtttcatgt

B-jararaca_D.07 gatgggaaggaattgtttgctccacttcccattcatagatacggtagtaatgtttcatgt

****** *************** ** * ********** ****** * *********

Exon 5

Ser Cys

B-atrox ttgatttttctcaacagGGTGAC**TCT**GGGGGACCCCTCATC**TGT**AATGGACAATTCCAGG

C-horridus ttgatttttctcaacagGGTGAC**TCT**GGGGGACCGCTCATC**TGT**AATGGAGAAATCCAGG

C-viridis ttgatttttctcaacagTCTGAC**TCT**GGGGGACCCCTCATC**TGT**AATGGACAATTCCAGG

P-mucrosquamatus ttgatttttctcaacagGGTGAC**TCT**GGGGGACCCCTCATC**TGT**AATGGACAATTCCAGG

B-jararaca_I.3.1 ttgatttttctcaacagGGTGAC**TCT**GGGGGACCCCTCATC**TGT**AATGGAACATTCCAGG

B-jararaca_D.10 ttgatttttctcaacagGGTGAC**TCT**GGGGGACCCCTCATC**TGT**AATGGACAATTCCAGG

B-jararaca_D.07 ttgatttttctcaacagGGTGAC**TCT**GGGGGACCCCTCATC**TGT**AATGGACAATTCCAGG

***************** *************** *************** * ******

Cys

B-atrox GCATTTTATCTTGGGGAAGTGATCCC**TGT**GCCGAACCGCGTAAGCCTGCCTTCTACACCA

C-horridus GCATTTTATCTGTGGGGGGCGATCCT**TGT**GCCCAATCTCATGTGCCTGCCCTCTACATCA

C-viridis GCATTTTATCTTGGGGAGACGATCCT**TGT**GCCCAACCGCATAAGCCTGGTGTCTACACCA

P-mucrosquamatus GTATTGTATCTTGGGGAGGCGATCCT**TGT**GCCCAACCTCGTGAGCCTGGCGTCTACACCA

B-jararaca_I.3.1 GCATTGTATCTTGGGGAGGTAAAGTC**TGT**GCCCGACCGCGTAAGCCTGCCCTCTACACCA

B-jararaca_D.10 GCATTTTATCTTGGGGAGGTATTCCC**TGT**GCCCAACCGCGTAAGCCTGCCTTCTACACCA

B-jararaca_D.07 GCATTTTATCTTGGGGAGGTATTCCC**TGT**GCCCAACCGCGTAAGCCTGCCTTCTACACCA

* *** ***** *** ****** * * * * ***** ****** **

Cys

B-atrox AGGTCTTTGATTATCTTCCCTGGATCCAGAGCATTATTGCAGGAAATAAAACTGCGACT**T**

C-horridus AGGTCTTTGATTATACTGAGTGGATCCAGAGCATTATTACAGGAAATACAGCTGCAACT**T**

C-viridis AGGTCTTTGATCATCTTGACTGGATCCAGAGCATTATTGCAGGAAATACAGATGCAACT**T**

P-mucrosquamatus ATGTCTTCGATCATCTTGACTGGATCAAGGGCATTATTGCAGGAAATACAGATGTAACC**T**

B-jararaca_I.3.1 AGGTCTTTGATTATCTTCCCTGGATCCAGAGCATTATTGCAGGAAATAAAACTGCGACT**T**

B-jararaca_D.10 AGGTCTTTGATTATCTTCCCTGGATCCAGAGCATTATTGCAGGAAATACAACTGCGACT**T**

B-jararaca_D.07 AGGTCTTTGATTATCTTCCCTGGATCCAGAGCATTATTGCAGGAAATACAACTGCGACT**T**

* ***** *** ** * ****** ** ******** ********* * ** ** *

Stop

B-atrox **GC**CC---G*TGA*----------

C-horridus **GC**CCCCCA*TGA*----------

C-viridis **GC**CCCTC-TGTAAACTTT*TGA*

P-mucrosquamatus **GC**CCCCTG*TGA*----------

B-jararaca_I.3.1 **GC**CCCCCG*TGA*----------

B-jararaca_D.10 **GC**CCCCCG*TGA*----------

B-jararaca_D.07 **GC**CCCCCG*TGA*----------

**** **
