# Supplementary material for: The absence of thrombin-like activity in Bothrops erythromelas venom is due to the deletion of the snake venom thrombin-like enzyme gene
Source: PLoS One. 2021 Apr 27;16(4):e0248901. doi: 10.1371/journal.pone.0248901 (PMC8078745; doi:10.1371/journal.pone.0248901)
Supplement: S1 Table — Sequences used in Datamonkey analysis, only the coding sequences without stop codons were analyzed. (DOCX) [file pone.0248901.s002.docx]

**S1 Table. Sequences used in selection analysis.**

| **Snake** | **Genbank accession number** |
| --- | --- |
| *Bothrops atrox* | X12747 |
| *Bothrops jararaca* | MT547769 |
| *Crotalus adamanteus* | JU173725, JU173722, JU173727, JU173723, JU173733, JU173737, JU173732 |
| *Crotalus horridus* | LVCR01039842 |
| *Crotalus viridis* | PDHV02000010 |
| *Macrovipera lebetinus* | GU570565, GU570567, AJ251153 |
| *Notechis scutatus* | XM026666390, XM026666391, XM026666392 |
| *Ophiophagus hannah* | EF080837, |
| *Philodryas_olfersii* | DQ139921, DQ139920, DQ912657, |
| *Protobothrops flavoviridis* | D67080, AB985230, AB848162, D67078 |
| *Protobothrops mucrosquamatus* | XM015816072, XM015816071, BCNE02033937, NM001323247, XM015816074, XM015816076 |
| *Trimeresurus gramineus* | D67083, D67084, D67082, D67085, D67081 |
