# Supplementary material for: The absence of thrombin-like activity in Bothrops erythromelas venom is due to the deletion of the snake venom thrombin-like enzyme gene
Source: PLoS One. 2021 Apr 27;16(4):e0248901. doi: 10.1371/journal.pone.0248901 (PMC8078745; doi:10.1371/journal.pone.0248901)
Supplement: S2 Table — M13 primers are complementary to borders of plasmid vector. BE primers are complementary to Bothrops erythromelas sequences. BJ primers are complementary to Bothrops jararaca sequences. (DOCX) [file pone.0248901.s003.docx]

**S2 Table.** **Primers using in Sanger sequencing.**

| **Primer** | **5’ – 3’ sequence** |
| --- | --- |
| M13-fw | TGTAAAACGACGGCCAGTG |
| M13-rev | GGAAACAGCTATGACCATG |
| BE-fw2 | TAGTCTTGTGTGGCTTCATTAC |
| BE-rev2 | CTTTGCCTCCTTCCAGGATAC |
| BE-fw3 | CCTTGGTCAAGAGATGGGAAATA |
| BE-rev3 | GGGAGGACAAATGAGGTAGAAA |
| BJ-fw2 | GAGATGGCCATTGATGTTTGTC |
| BJ-rv2 | CCACATGTATCTATGCCTCCTT |
| BJ-fw3 | ACAGAACTGATCTTGCATCCC |
| BJ-rv3 | TCCCTTGGTTGGAGACAATG |
| BJ-fw4 | AGATCAGACTCTGGACCAAATG |
| BJ-rv4 | AAGGAGTGAAAGTGGAGAAGAG |
| BJ-FW5 | GCTTTCGGATTATGGCCTTTG |
| BJ-rv5 | TGCTCTAGCCACACCTATCT |
| BJ-FW6 | GTGTCCCACAGTCATCTGTTAC |
| BJ-rv6 | CTACCGTCAGGAGTTGTGATTG |
| BJ-fw7 | CAGCCTTACTTGGAACATCCT |
| BJ-rv7 | AAGCTGGGTCTGAGAGTATAGA |
| BJ-fw8 | CGCCCAAATCTTATCACCACTA |
| BJ-rv8 | TAACGTGTGCCAGTGTGTTC |
| BJ-fw9 | GCACTCTCAGCTTTGGCTTAT |
| BJ-rv9 | AAGGGCCATAACCTAACTTCTC |

M13 primers hybridize to the plasmid vector's borders, BE primers to *Bothrops erythromelas* sequences, and BJ primers to *Bothrops jararaca* sequences.
